# Corilagin functionalized decellularized extracellular matrix as artificial blood vessels with improved endothelialization and anti-inflammation by reactive oxygen species scavenging

**DOI:** 10.1093/rb/rbae074

**Published:** 2024-07-01

**Authors:** Xu Wang, Hanmei Fu, Huibin Wu, Xiaohua Peng, Xu Peng, Xixun Yu, Hui Liu, Junmei Wu, Ling Luo, Shan Yan, Xinglin Cheng, Xiong Zhou, Xiangyang Yuan

**Affiliations:** School of Acupuncture and Tuina, Chengdu University of Traditional Chinese Medicine, Chengdu 610075, China; School of Acupuncture and Tuina, Chengdu University of Traditional Chinese Medicine, Chengdu 610075, China; College of Science, Sichuan Agricultural University, Ya’an 625014, China; School of Acupuncture and Tuina, Chengdu University of Traditional Chinese Medicine, Chengdu 610075, China; College of Polymer Science and Engineering & Laboratory Animal Center, Sichuan University, Chengdu 610065, China; College of Polymer Science and Engineering & Laboratory Animal Center, Sichuan University, Chengdu 610065, China; School of Intelligent Medicine, Chengdu University of Traditional Chinese Medicine, Chengdu 610075, China; School of Acupuncture and Tuina, Chengdu University of Traditional Chinese Medicine, Chengdu 610075, China; College of Traditional Chinese Medicine, The University of Hong Kong, Hong Kong 999077, China; School of Acupuncture and Tuina, Chengdu University of Traditional Chinese Medicine, Chengdu 610075, China; School of Acupuncture and Tuina, Chengdu University of Traditional Chinese Medicine, Chengdu 610075, China; School of Acupuncture and Tuina, Chengdu University of Traditional Chinese Medicine, Chengdu 610075, China; Department of Biomedical Engineering, City University of Hong Kong, Hong Kong 999077, China; College of Science, Sichuan Agricultural University, Ya’an 625014, China

**Keywords:** corilagin-crosslinked blood vessels, ROS consumption, anti-inflammation

## Abstract

The performance of biological-originated blood vessels in clinical remains disappointing due to fast occlusion caused by acute thrombosis or long-standing inflammation. How to prevent rapid degradation and inhibit acute inflammation but maintain their high bioactivity is still a significant challenge. As a bioactive polyphenol in various traditional Chinese medicine, Corilagin (Cor) exhibits excellent anticoagulant, anti-inflammatory and rapid ROS consumption properties. Inspired by abundant supramolecular interactions in organisms, we selected it to crosslink tissues via purely H-bonds to simulate these natural interactions without introducing potential toxic aldehyde or carboxyl groups. Results show that 2 mg/ml was selected as the optimal Cor concentration to form a stable crosslinking network (FI > 95%) and effectively delay their degradation. Cor modification not only enhances ECs adhesion and monolayer function via accelerating VEGF and TGF-β secretion but also promotes macrophage transformation from pro-inflammatory M1 phenotype to anti-inflammatory M2 ones. *In vitro* and *ex-vivo* studies implied that Cor-crosslinked samples exhibited low platelet accumulation and decreased thrombin generation. *In vivo* evaluation further confirmed that Cor-introducing could effectively consume ROS, thus exhibiting rapid endothelialization, suppressed inflammation and reduced mineral deposition. Overall, Cor crosslinking provided a bright future for blood vessels’ long-term patency and adapted to various blood-contacting surfaces.

## Graphical abstract

**Figure:**
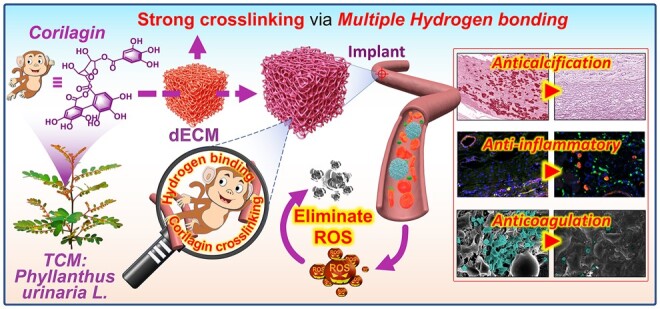

## Introduction

Vascular diseases remain the leading cause of death globally. Currently, replacing a damaged or occluded blood vessel with an artificial one to restore blood flow and prolong its patency period is still an effective solution [1, 2]. Attributing to abundant bioactive factors and appropriate micro-porous structures similar to natural vessels, natural-derived biological blood vessels have attracted more and more attention, and they have been used with increasing frequency for therapy [3, 4]. Unfortunately, their application is still limited by the serious immunogenic response and high transmission risk of diseases [5, 6]. To resolve these issues, various active molecules with multiple functional groups (e.g. aldehydes and epoxides) were introduced to construct a stable crosslinking network via covalent bonds [7–9]. However, they tend to react with local tissues and even alter vascular permeability. In addition, continuously dissolved molecules can freely penetrate cell membranes to cause significant cytotoxicity, resulting in serious local inflammation, which is considered a critical factor in the high incidence of thrombosis, intimal hyperplasia, and calcification [10].

Recently, polyphenols and their derivatives have been considered novel promising crosslinking reagents to treat vascular anastomotic inflammation and avoid acute thrombosis. Different from common covalent crosslinking, they build 3D networks through pure hydrogen binding without chemical structure change [11, 12]. That means the structural stability of the biological tissue-based scaffolds could be improved by additional active functional groups through supramolecular interactions, which already widely exist in biosystems, without permanent covalent bonding. These noncovalent interactions are more favorable for the sustained release of these polyhydroxylated active biomolecules to surrounding tissues.

**Figure 1.**
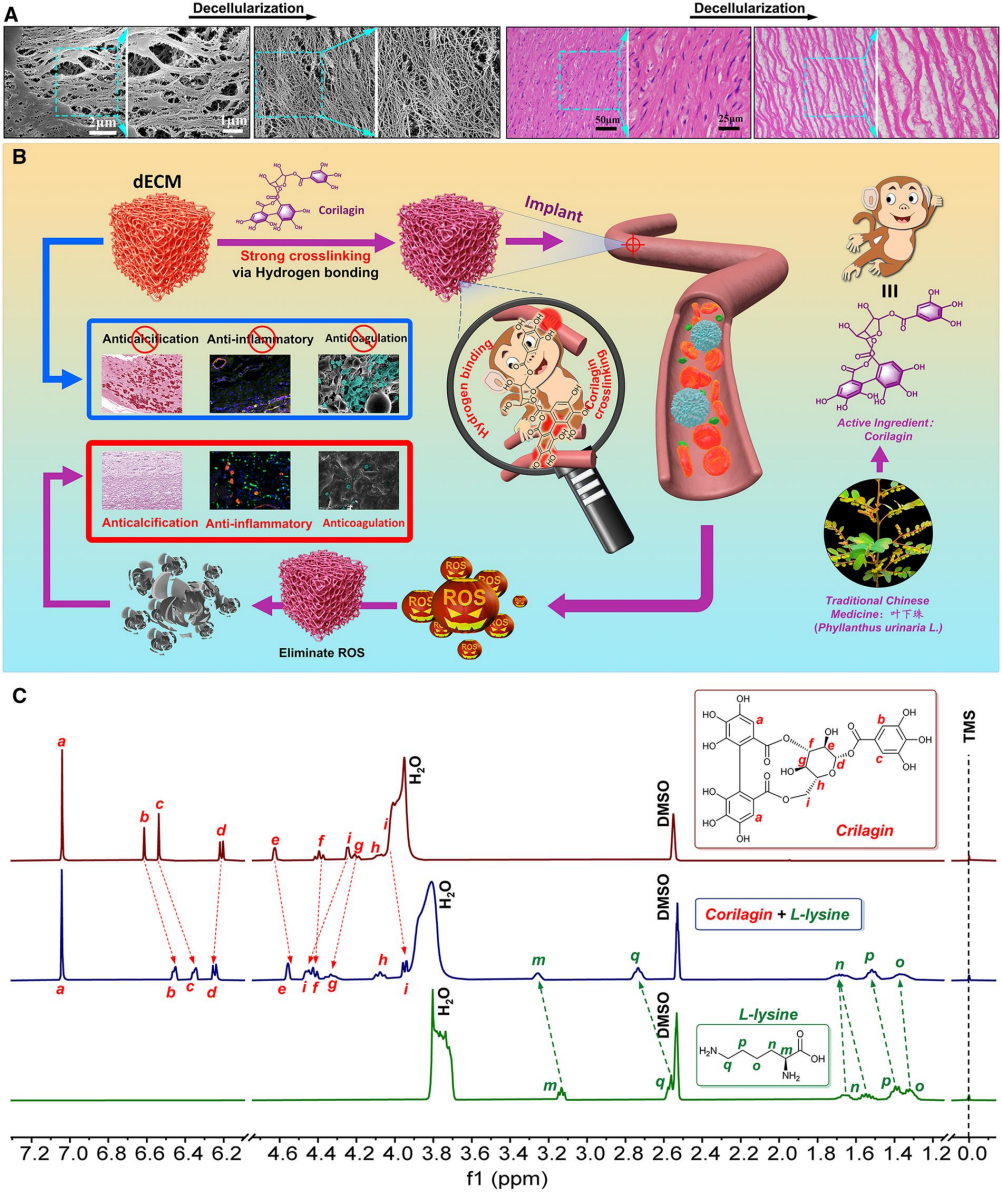
Characterization of Cor-crosslinked blood vessels. (**A**) Aortas before and after decellularization were observed by SEM and H&E stain (200 ×). (**B**) Schematic diagram of Cor crosslinking with multiple polyphenols for rapid ROS consumption. (**C**) ^1^H NMR analysis for H-bonding strength detection.

Corilagin (Cor) is a natural-derived bioactive polyphenolic compound extracted from traditional Chinese medicinal herbs, namely *Phyllanthus urinaria* L., or it could also obtained from *longan seeds* and *Phyllanthus emblicanine et al.* This important ingredient in TCM exhibited multiple functions such as astringency, anti-inflammatory and anticancer, making it a good anti-tumor, hepatoprotective and anti-inflammatory agent in biomedical applications [13, 14]. It can even be used for special treatment of fulminant myocarditis and encephalitis. As shown in Figure 1, Cor is composed of nine bioactive phenolic hydroxyl groups on three rigid benzenes, linked to a D-glucopyranose ring with ester bridges, which endowed it with a strong antioxidant capacity to avoid potential inflammatory risks caused by reactive oxygen species (ROS) release [15–17]. Furthermore, their promotion for endothelial cell (ECs) growth makes Cor a promising crosslinking reagent for blood vessel fixation. The unique chemical structure of Cor provides both abundant hydrogen binding donors (-OH) and receptors (oxygen atoms in both -OH and -COOR) for it to form strong interactions by multiple hydrogen bonds with surrounding tissues. It is just like a monkey being able to firmly grasp different branches with limbs and tails at the same time. Moreover, the out-oriented Ar-OH groups on these three benzene rings possessed specific angles, which ensured themselves as active sites to participate in rapid crosslinking without steric hindrance. At the same time, their rigid structure will also provide good mechanical strength for the final crosslinking. They can form dense H-bonds crosslinking on the surface of collagen fibers to avoid metalloproteinase attack, fiber denaturation or microscopic morphology changes [16, 18]. Besides, vascular substitutes treated by chemical crosslinking reagents often exhibit serious calcification drawbacks, which are closely related to elastic fiber destruction. Since the elastic fibers lack active sites for chemical modification, they are hard to stabilize via covalent interaction. In comparison, the Cor H-bonds construction could effectively protect them, which is beneficial for avoiding tissue calcification and suppressing chronic inflammation [12, 16, 19]. Thus, Cor-crosslinking could effectively improve tissue stability via supramolecular interactions (H-bonds) similar to natural organisms rather than additional chemical reactions.

It has been found that dialdehyde polysaccharide derivatives could rapidly crosslink biological tissues and endow them with tunable mechanical properties matched to native blood vessels in our previous studies [7, 20, 21]. However, the main barrier in developing dECM small-diameter vascular grafts is acute thrombosis and intimal hyperplasia promoted by inflammation at anastomotic sites. We have investigated surface modification strategies to overcome it, including a reduction for post-processing, peptide or protein coating and bioactive ceramic particle doping [22–24]. However, how to stabilize the modification and maintain long-term patency is still a great challenge.

In the present study, we employed Cor without any supplementary toxic functional groups as an active ingredient as well as a crosslinking agent for dECM, and the modification was carried out under mild aqueous conditions. We expected that Cor could consume ROS to exert anti-inflammatory effects and establish a favorable microenvironment locally, which is a benefit for promoting rapid endothelialization and avoiding acute thrombus at vascular anastomosis sites. Additionally, profiting from their abundant H-bond crosslinking, elastic fibers could also be well protected, and it is endowed with the potential to resist calcification and maintain long-term patency.

## Materials and methods

Except the information of materials and reagents, fixation index (FI) determination, weight loss detection in enzymolysis, cell culture and seeding, animal experiments, accumulated Cor release and other detailed experimental processes that were descripted in the Supplementary Information. General experiments carried out for this work were described as below.

### Decellularization and Cor-crosslinking

Fresh porcine aortas purchased from local abattoirs were employed as raw materials for decellularization. Briefly, native aortas were incubated with phosphate-buffered saline (Supplementary Table S1) (PBS, 0.1 M, pH 7.2) containing trypsin (0.25%, w/v) and EDTA (0.02%, w/w) for 24 h at room temperature. Then, the resulting tissues were placed into a 1% Triton-100/PBS buffer for another 24 h to eliminate cell lysis. Subsequently, 0.02 mg/ml RNase A and 0.2 mg/ml DNase I were simultaneously introduced and stirred for 24 h under an ambient atmosphere [19, 25]. Then, native and decellularized aortas were fixed for hematoxylin-eosin (H&E) stain and scanning electron microscope (SEM) observation.

To crosslink tissues, decellularized samples were immersed in Cor/DMSO solution with a series of gradient concentrations (0.2, 0.5, 1.0, 2.0, 5.0 and 10 mg/ml). A constant shaking was necessary to make the Cor completely diffuse into fibers (Supplementary Figure S1). Then, they were kept in a cabinet with controlled temperature (37°C) and humidity (60–70%) until fully crosslinked [19, 26]. Finally, these crosslinked samples were rinsed in PBS several times to remove the residual Cor, and samples crosslinked by 0.625% glutaraldehyde (GA) were taken as the positive control. Free amine content after crosslinking was estimated via ninhydrin assay. Meanwhile, a further quantitative detection for H-bonds number and strength was revealed by ^1^H NMR analysis.

### Characterization of crosslinking stability

#### Collagenase and elastase degradation

To evaluate the resistance against enzyme degradation of Cor-crosslinked samples, the reported method was applied with slight modification. After complete crosslinking, samples (*n* = 5) were incubated with Tris buffer (0.1 M, pH = 7.4) containing collagenase I (125 U/ml) and elastase (30 U/ml) for 24 h at 37°C [18]. Followed by termination via adding 10 mM EDTA, the residual samples were lyophilized to calculate the weight loss rate or fixed in 4% paraformaldehyde (PFA) for fiber micro-morphology observation.

#### Biomechanical strength

dECM samples were cut along the collagen fiber direction to 15 mm × 5 mm sections, and then the biomechanical strength of various samples (*n* = 5) was determined via Instron material testing machine (Instron Co., USA) with a 100-N load cell at a constant speed of 10 mm/min [20]. Once fracture occurred, the first decrease in the load of the tested sample was recorded as ultimate tensile stress, and relevant Young’s modulus was obtained by measuring the slope of the stress–strain curve in the high-strain region.

### *In vitro* hemocompatibility

For platelet adhesion, anti-coagulated blood with sodium citrate was centrifuged at 1500 rpm for 10 min to acquire the platelet-rich plasma (PRP). For hemolysis detection, anti-coagulated blood with citric dextrose was centrifuged at 500 rpm for 10 min and repeatedly washed with sterile PBS to obtain red blood cell (RBC) suspension.

#### Platelet adhesion assay

Various samples were immersed into PRP (300 μl/well) for 2 h under constant wobbling at 240 rpm [23, 27]. Subsequently, all samples were repeatedly washed with PBS to remove unattached platelets and fixed with 2.5% GA for SEM observation. Meanwhile, their quantitative determination was achieved by lactate dehydrogenase (LDH) assay via reading their absorbance at 490 nm.

#### Hemolysis detection

Each sample (*n* = 6) was ground to powder and dispersed in 800 μl PBS to form gradient-concentration (5, 10, 25, 50, 100, 200, 400, 1000, 2000 and 4000 μg/ml) suspension. Then, 200 μl RBCs suspension was added to each tube and co-incubated at 37°C for 4 h [28]. Isotonic PBS and distilled water were used as the negative and positive control, respectively. After that, the supernatant was obtained via centrifuging at 10 000 g for 3 min, and its absorbance was detected at 540 nm. The hemolysis ratio was calculated as follows:


$$\text{Hemolysis rate}\;\left(\%\right)=\left(A_{\text{sample}}-A_{\text{negative}}\right)/\left(A_{\text{positive}\;}-A_{\text{negative}}\right)\times 100\%$$

### Cytocompatibility analysis

Human umbilical vein endothelial cells (HUVECs) and RAW 264.7 purchased from West China Hospital were introduced to evaluate the biocompatibility of Cor-crosslinked samples.

For HUVECs: They were sterilized via ultraviolet irradiation and then seeded with cells in a density of 5 × 10^4^ cells/cm^2^ [25, 29]. After a pre-determined period of incubation, cell adhesion and function were analyzed:

For RAW 264.7: After the digestive procedure, 5 × 10^4^ cells/ml mouse-derived macrophage RAW264.7 was seeded into extracts of various samples in a 24-well culture plate. Then, 1 μg/ml lipopolysaccharide (LPS) was added to each well, to induce macrophage polarization to M1 phenotype for 12 h. Then, cell morphology and cytoskeleton staining were collected after 4 days of co-incubation, and the secretion level of inflammatory-related factors (IL-6, IL-10, TGF-β) in their supernatants was measured through ELISA kit.

#### Cytoskeleton staining

After 4 days co-culture, cells were fixed with 4% PFA overnight, permeabilized with 0.1% Triton X-100, and blocked with 1% bovine serum protein. Subsequently, the cells were stained with fluorescein isothiocyanate-phalloidin (Solarbio) and 4′,6-diamidino-2-phenylindole (Solarbio) for 30 min to observe cytoskeleton and cell nucleus. Cells’ adhesion and viability were visualized with a confocal laser scanning microscope (CLSM, TSC SP8) [30].

#### SEM observation

Adhered cells on Cor-modified tissues were fixed with 2.5% GA/PBS buffer. Then, to avoid cellular morphological changes, tissues were dehydrated in increasing ethanol concentration and critical point-dried for SEM observation.

#### ELISA assay

After 3 days’ co-culture, the supernatant of each sample was collected via centrifuging at 14 000 rpm for 5 min. Secretion levels of VEGF and TGF-β from HUVECs, as well as IL-6, IL-10 and TGF-β from RAW, were evaluated through double ligand enzyme-linked immune sorbent assay (ELISA) according to manufacturer’s instructions [5].

### *In vitro* antioxidant efficiency

The antioxidant efficiency of various Cor-crosslinked samples was evaluated by DPPH free radical scavenging assay and ROS assay, respectively.

In the DPPH assay, various samples (*n* = 3) were ground to powder and dispersed in 3.0 ml ethanol to form gradient-concentration suspension (0.5, 1.0, 3.0, 5.0, 10 mg/ml). Then, 100 μl DPPH was added to each well and maintained a photophobic incubation for 1.5 h at 37°C. After centrifugation, the OD value of the supernatant was read at 517 nm, and the DPPH scavenging (%) was calculated as the following formula [31]:


$$\text{DPPH scavenging}\;\left(\%\right)=\left(A_{\text{Blank}}-A_{\text{Sample}}\right)/\left(A_{\text{Blank}}\right)\times 100\%$$

The intracellular ROS scavenging abilities of Cor -crosslinked samples were evaluated in real-time via ROS assay. Briefly, after ECs were seeded in 5 × 10^5^ cells/well density and incubated for 12 h, samples suspension containing 1 μg/ml Rosup was added and maintained for 30 min. Then, they were photographed by CLSM within 4 h.

### Tissues implantation

All animal protocols were approved by the Animal Care and Use Committee of Sichuan University. Male SD (4-month) rats were used in our subcutaneous implantation (ethical approval number: 2024020103e). Briefly, after SD rats were anesthetized by inhaling isoflurane gas, two symmetrical and longitudinal surgical incisions were made on both sides of their back. Then, the crosslinked samples above were implanted into these subcutaneous pockets for further revascularization, anti-oxidation, anti-inflammation and anti-calcification evaluation *in vitro* [23].

#### Histology and immunohistochemistry

The implanted sample, together with adherent tissue, was collected and fixed in formalin overnight for histological analysis. Critically, samples were serially dehydrated, parafilm embedded, and sectioned. To evaluate the anastomosis regeneration, H&E staining, and immunohistochemistry staining for CD31 and α-SMA were utilized to semi-quantitatively detect vascular functions. Meanwhile, IL-6, IL-10, TNF-α and TGF-β staining was carried out to evaluate their anti-inflammatory properties.

#### Fluorescent mark and immunofluorescence

ROS could be marked by fluorescent probes-2′,7′-dichlorofluorescent yellow diacetate (DCFH-DA, Beyotime, China), and its concentration was recorded according to fluorescence intensity. Additionally, immunofluorescence for CD68, CD206, and CD86 expression was detected via flow cytometry assay (FCA) apparatus (Merck Millipore, Germany) to further evaluate their anti-inflammatory mechanism.

#### Calcium analysis

Alizarin red, Von Kossa, OCN and OPN stains could simultaneously visualize calcium deposition morphology after tissues’ long-term (12 weeks) implantation.

### Statistical analysis

The data with parallel samples mentioned above were obtained from at least three repeated experiments. SPSS (v19.0) was applied for statistical analysis. Quantitative data were depicted as a mean value with its standard deviation (mean ±SD, *n* = 3/5). To determine the differences in measured properties of parallel experimental groups, a one-way analysis of variance (one-way ANOVA) was performed, and their statistical significance was set at *P* < 0.05.

## Results and discussion

### Decellularization and corilagin-crosslinking

Natural-derived aortas are considered to be the future trend of blood vessel substitutes. However, the native tissues should be pre-decellularized to avoid immunological recognition and progressive calcification [5]. As shown in Figure 1A, no cellular components were detected in the filamentary pores of tissues via H&E staining, which indicated a complete decellularization and scarce immunological recognition. A similar result was also confirmed by SEM observation: Naturally, curly fibers were still regularly distributed without being thinned and broken after decellularization, which is beneficial to provide a suitable environment for cell adhesion and proliferation [18].

Then, a stable crosslinking network is rapidly constructed, and the schematic diagram of the Cor-fixation process is exhibited in Figure 1B. As presented, Cor is a molecular platform with nine phenol groups on the rigid terminal. These phenol groups are regarded as the main reaction sites and all point outward, easy to form interwoven H-bonds with -NH_2_ on tissues and avoid steric hindrance. In addition, the D-glucopyranose ring in the center endowed molecules with a certain flexibility, which makes it easy to bridge between adjacent fibers through swinging and thus improves mechanical strength. More importantly, as a hydrophilic small molecule substance, Cor is easy to diffuse into fibers and form an outer-inner uniform crosslinking network. This process is mainly realized by H-bonding without introducing covalent linkages, which is beneficial for preventing serious cytotoxicity and side reactions.

The original acellular matrix makes it difficult to achieve fully satisfactory performance, while Cor modification seems able to overcome its shortcomings. They can form multiple noncovalent interactions rapidly with decellularized tissues to achieve stable crosslinking, as well as maintain their sufficient active functional groups to provide antioxidant, anticoagulant and organization-friendly properties [17]. Generally, the ^1^H NMR spectrum could analyze intramolecular interactions for accurate chemical structure identification. However, these proteins (including the critical ones such as elastin) are solid in fibers, and it is hard to identify their noncovalent interactions in deuterated solvents directly. Therefore, we selected L-lysine, the main amino acid component in elastin, as the model molecule for ^1^H NMR analysis [32]. As shown in Figure 1C, the ^1^H NMR signal of H_b_ and H_c_ on Cor shifted upfield from 6.61 ppm and 6.54 ppm to 6.45 and 6.35 ppm, indicating a stronger shielding effect than L-lysine alone. This results from an increased electron density for hydrogen binding between hydrogen bonding acceptor -NH_2_ (L-lysine) and donor Ar-OH (Cor). At the same time, the ^1^H NMR signal of H_m_ and H_q_ shifted downfield to 3.26 ppm and 2.73 ppm from 3.13 ppm and 2.57 ppm. In addition, signal peaks of protons o and p also shifted slightly toward the lower field, which indicated a reduced shielding and could also be attributed to hydrogen binding. It means that Cor can form strong H-bonding interactions with the amino acid structural units of proteins, which is conducive to its stable binding in the fiber network. Meanwhile, due to the noncovalent interaction of hydrogen bonds, the slow release of Cor can still be maintained and has the potential to exert multiple drug effects *in vivo*. A similar result was confirmed in the ninhydrin assay (Supplementary Figure S2). Free amines in tissues, which have a risk of triggering immune responses, are almost rapidly consumed by Cor crosslinking in concentration gradient.

### Characterization of corilagin-crosslinking stability

Although natural-derived tissues exhibited high porosity fitting for cell adhesion and proliferation, their trace-residual immunological rejection and rapid degradation are still the main barriers to clinical application. Thus, Cor crosslinking was introduced to overcome these problems [5].

**Figure 2.**
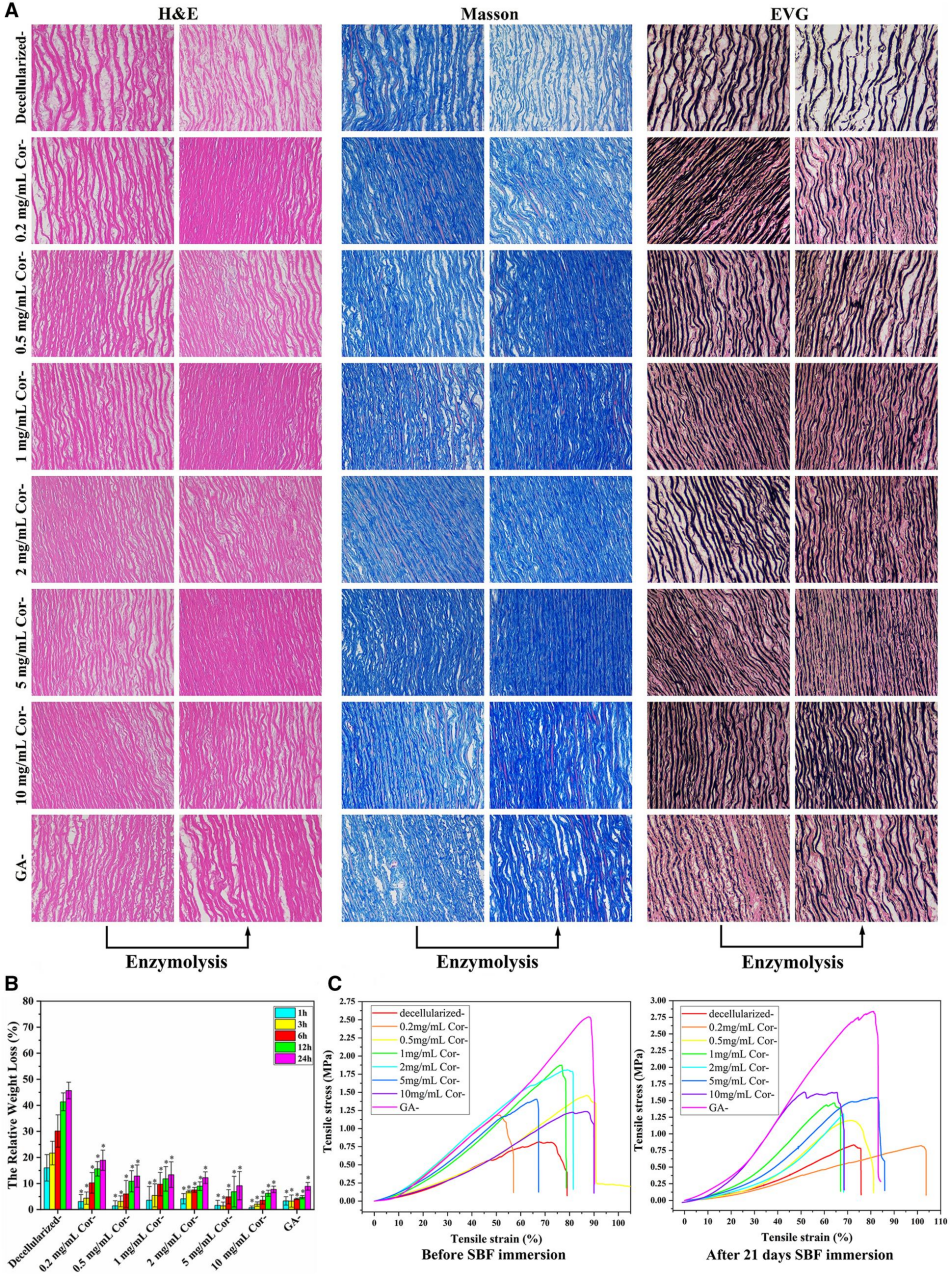
Tissue stability after Cor-crosslinking. (**A**) Modified aortas observed by H&E, Masson and EVG stain (200 ×) before and after 24 h digestion. (**B**) Relative weight of samples after collagenase and elastase digestion (* means significant difference compared to decellularized sample, *P < 0.05), all experiments were carried out at least 3 times. (**C**) Stress–strain curves of aortas enhanced by Cor-crosslinking. Decellularized- stands for decellularized blood vessel groups without modification, GA- and Cor- stand for GA and Cor cross-linked groups derived from decellularized samples, respectively.

It is necessary to determine whether Cor crosslinking could form a 3D network to improve collagen and elastin stability. Significant-enhanced mechanical strength and well-preserved ultrastructure after enzymolysis are considered two typical factors for tissue successful modification [25]. In the enzymolysis digestion assay (Supplementary Table S1), decellularized tissues suffered exhaustive degradation. Its weight loss percentage is up to 46.12%, and inner fibers turned fuzzy and mussy, gradually losing elasticity and load-bearing capability. As expected, all crosslinked samples showed a significantly lower weight loss than decellularized ones (Figure 2B and Supplementary Figure S3), which indicated an effective crosslinking network formed, consistent with FI measurement. While 0.2 and 0.5 mg/ml Cor-crosslinked samples exhibited much more weight loss than GA-fixed ones (8.94%), suggesting their low crosslinking density made it hard to provide adequate protection for fibers. Once the concentration of Cor exceeds 2 mg/ml, the tissue framework, including porous ultrastructure in the cross-section, will remain intact with only 3–8% weight loss. In particular, Figure 2A revealed that Cor crosslinking could simultaneously preserve collagen and elastin fibers, while in GA control, elastic fibers were seriously destroyed, and their porous structure disappeared. That may be because the high density of H-bonding interaction in Cor not only obstructs most enzyme-attacked sites in collagen but also provides a barrier to protect elastic fibers, which is also critical to block mineral nucleation sites of elastin to avoid calcification. On the contrary, chemical crosslinking could only form a stable network with high reactivity amine groups (lysine and hydroxylysine) in collagen. In contrast, elastin is mainly composed of hydrophobic amino acids and has scarce reactive groups, which makes it fail to be stabilized via chemical crosslinking. Additionally, Cor-crosslinked tissues exhibited a much higher porosity and a larger pore size, which is beneficial to cell growth. At the same time, the porous structure in GA-ones completely disappeared and became a dense structure to limit cell infiltration and even delay wound regeneration. When it was challenged with elastase, an evident weight loss was observed. While, thanks to the biodegradability of tissues, Cor molecules will be gradually released, with the potential to exert biological activity at vascular anastomosis to maintain blood vessels long-term patency (Supplementary Figure S4).

Buckling occurring at sharp bending or furcated sites may cause mechanical fatigue and consequent vascular deformation. Fiber integrity of implanted vessels plays a primary role in mechanical support for vasoconstriction/dilation function [7]. As shown in Figure 2C and Supplementary Table S2, their stress–strain curve was obtained to evaluate the biomechanical strength. Once fixed by Cor, the ultimate tensile stress of tissues was significantly increased. Particularly, it exhibited a significant concentration dependence and reached a peak at 2 mg/ml concentration (1.76 MPa), comparable to that of the GA-fixed one. With further increase in Cor concentration, no significant difference was presented in tensile stress. Moreover, the biological tissues crosslinked by Cor still maintain high mechanical strength after 21 days immersion in simulated body fluids (SBF) (Supplementary Table S3). In addition, the samples that suffered from SBF immersion have an obvious yield stage. It implied that a small part of crosslinking bonds was released after long-term immersion, which induced their relative slip between long polymer chains. In a word, a compact H-bonding interaction formed within collagen and elastic fibers after Cor-crosslinking, and this modification provided adequate mechanical functions for blood vessels fitting for patient survival. This result demonstrated that Cor crosslinking could improve the stability of tissues by forming an enormous number of H-bonds, whose effect is equivalent to that of chemical modification. In addition, the crosslinking effect of Cor showed a strong dose-dependent manner: With the concentration of Cor increased, the biomechanical strength of relevant samples gradually increased, indicating improved structural stability.

### *In vitro* antioxidant efficiency

**Figure 3.**
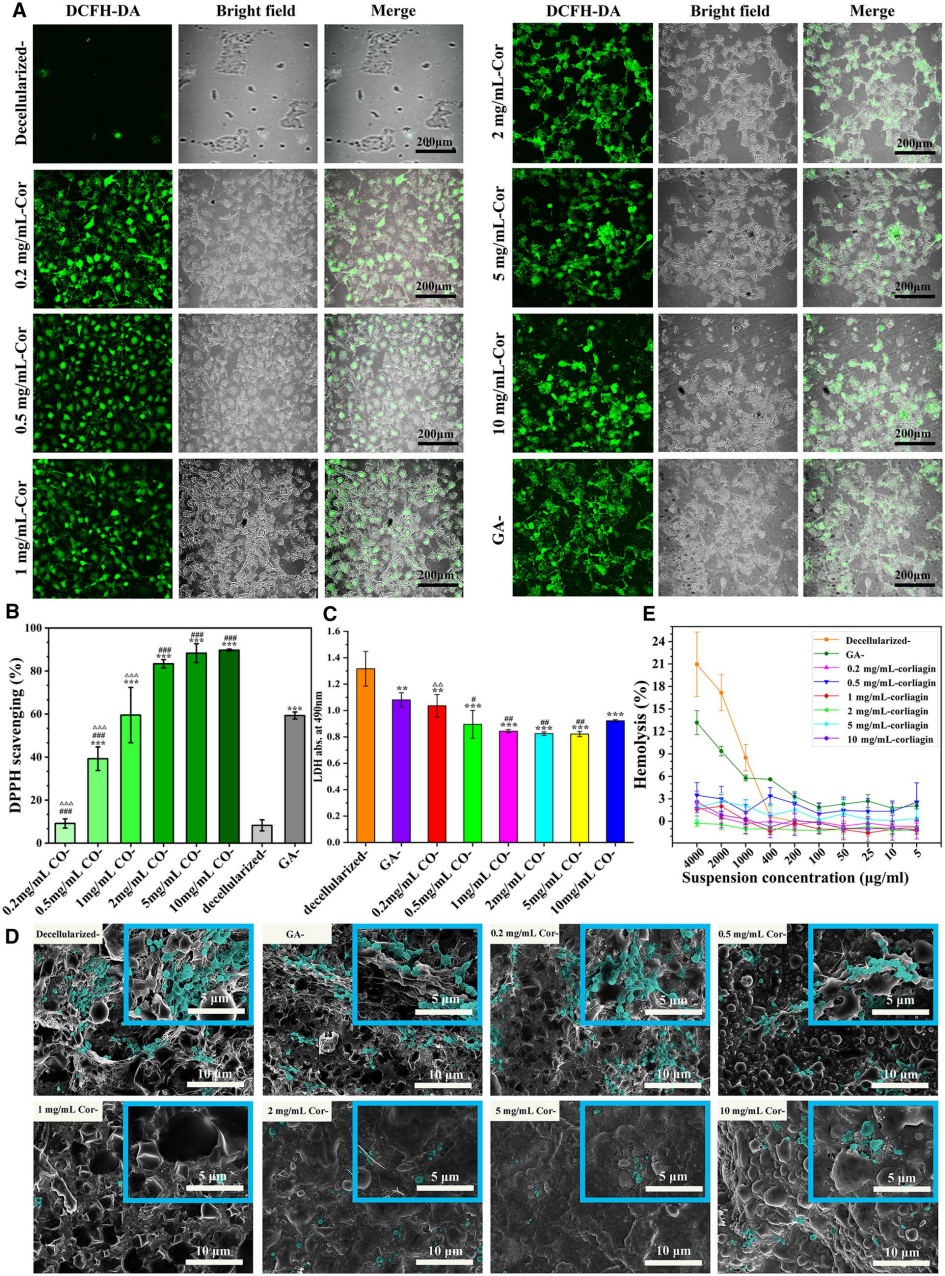
*In vitro* antioxidant efficiency and hemocompatibility. (**A**) Intracellular ROS level reduction and (**B**) DPPH scavenging abilities of Cor-crosslinked tissues. (**C**) Platelet adhesion was measured by LDH assay. Among them, *P < 0.05, **P < 0.01, ***P < 0.001 compared to decellularized group; #*P* < 0.05, ##*P* < 0.01, ###*P* < 0.001 compared to GA-crosslinking group; △*P* < 0.05, △△*P* < 0.01, △△△*P* < 0.001 compared to 2 mg/ml Cor-group. (**D**) SEM observation for platelet morphology (colored with green). (**E**) HR values of samples incubated with RBCs at 37°C for 4 h. All data in (B), (C) and (E) were obtained from at least 3 repeated experiments.

Excessive ROS in vascular anastomosis is the main reason for acute or chronic inflammations, even causing endothelial damage and persistent local inflammation. For newly implanted vessels, repetitive infection and immune rejection can lead to high ROS accumulation and form an oxidative stress microenvironment [33]. Thus, a polyphenolic compound (Cor) was introduced to endow blood vessels with good anti-oxidation. As displayed in Figure 3B, owning to large numbers of free phenol groups, Cor-crosslinked samples exhibited a significant anti-oxidation effect, and their radical scavenging efficiency was more than 80%. Additionally, its oxidation resistance was concentration-dependent. The radical scavenging level gradually promoted with Cor concentration increasing and maintained a constant level once the Cor concentration was over 2 mg/ml. Furthermore, the ROS assay was employed to test the intracellular ROS scavenging capability. As shown in Figure 3A, the fluorescence intensity of ECs on Cor-crosslinked tissues decreased with Cor-concentration increasing. Then, fluorescence quenching occurred on 2 mg/ml Cor-crosslinked samples, similar to the ROS–untreated control. That may be because there is still a large number of free phenol groups left after tissue crosslinking, and this exposed Cor could play a critical antioxidant role via scavenging free radicals, chelating metal cations, and inhibiting oxidase activity.

### *In vitro* hemocompatibility

For long-term blood contact, hemocompatibility is an important consideration for blood vessels to prevent acute thrombosis formation or even implantation failure [29, 30]. As exhibited in Figure 3D, there are many platelets adhered to decellularized-, GA- and Cor samples at low concentrations (0.25 and 0.5 mg/ml). Especially, the morphology of platelets on them tends to spread irregularly with several pseudopods, implying high activation. Mean only sporadic and round-shaped platelets were visible on higher concentrations of Cor (1, 2, 5 and 10 mg/ml) fixed ones. It indicated that samples crosslinked by Cor in appropriate concentration can inhibit platelets aggregation and thrombin generation, thus having better hemocompatibility. A similar result was also obtained in the LDH assay in Figure 3C. The OD value of the GA-crosslinked one is up to 1.1, which is significantly higher than the Cor-modified one. The reduced platelet adhesion on Cor-crosslinked samples could be explained as follows: Firstly, there are large amounts of galloyl groups covered on their surface, which have no significant effect on improving the adsorption capacity of fibrinogen (Fgn), but they might have changed their conformation that is beneficial to platelets adhesion. (The exposure degree of γ-chain, functioned for Fgn-adsorbing, is positively correlated with platelets adhesion.) It can be inferred that, the more galloyl groups existed, the lower the exposure degree of γ-chain exhibited, and the fewer platelets adhered. Galloyl groups could form strong hydrogen bonds with Fgn to prevent their conformation change and make the γ-chain receptors hidden. As a result, the adhesion of platelets would be effectively rejected. Secondly, their large number of polyphenols can eliminate ROS and promote anti-inflammatory factors secretion. It could also inhibit coagulation activation indirectly, thereby reducing platelet adhesion [15, 34]. Relatively, it is highly likely that the pro-coagulability of decellularized- and GA-fixed samples was mainly caused by the large amounts of residual carboxyl, amino and even toxic aldehyde groups on tissues, leaving a potential risk of charge aggregation and further activation of platelets. Further effects of Cor-crosslinking on clotting activity were assayed (Supplementary Figures S5 and S6), which indicated Cor crosslinking could significantly prolong APTT, TT, PT and decreased FIB. Thus, Cor-crosslinking could prevent samples from clotting activity by residual tissue factors, and the phenol groups in Cor are safe for blood vessel crosslinking. It exhibited no complement-activating activity through alternative pathways.

Besides, Cor-crosslinking could effectively avoid tissue rapid degradation and biological environmental changes, preventing serious damage to erythrocyte cells in long-term blood-contacting. As Figures 3E and Supplementary Figure S7 displayed, the HR values of decellularized- and GA-fixed samples were up to 21.02% and 13.09%, which were far beyond the acceptable value (5%) for clinical application. On the contrary, Cor-crosslinked samples presented lower HR values and 2 mg/ml-Cor crosslinked possessed the lowest one (0.03%). It indicated that bioactive polyphenol compounds introduced would prevent RBCs destruction, keeping them within international safety standards for vascular transplantation.

### Endothelial adherence and functions

Rapid re-endothelialization on blood vessels is critical to maintain their mechanical stability for long-term application. Furthermore, it could also inhibit thrombosis and neointimal hyperplasia [23, 35]. To evaluate the endothelialization potential of Cor-crosslinked samples, the morphology, adhesion, and proliferation of ECs on them were detected. As the CCK-8 assay displayed in Supplementary Figure S8, the optical density (OD) value of Cor-crosslinked groups is higher than that of blank control, which revealed their better cell viability and almost non-existent cytotoxicity. The OD value reached the maximum at 2 mg/ml of Cor, but when the concentration further increasing, the OD value decreased. On the contrary, decellularized- and GA-fixed samples exhibited a notable inhibition for cell proliferation.

**Figure 4.**
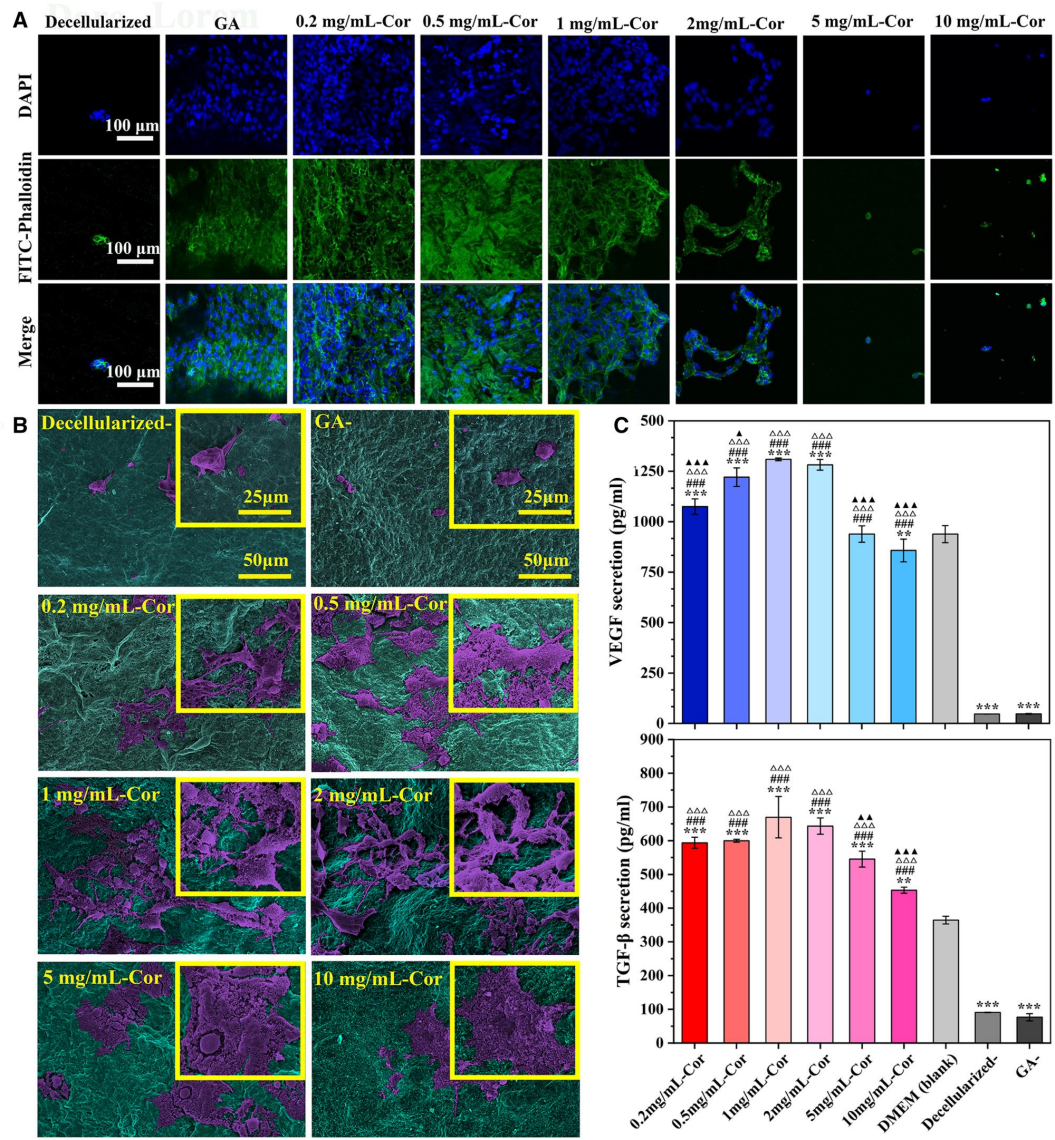
Endothelial adherence and function analysis. (**A**) Cytoskeleton staining and (**B**) SEM observation of ECs co-cultured with various concentrations of Cor-crosslinked tissues for 4 days. (**C**) VEGF and TGF-β secretion in supernatant from adherent ECs. All these data were obtained from at least three repeated experiments. Among them, *P < 0.05, **P < 0.01, ***P < 0.001 compared to the DMEM (blank) group; #*P* < 0.05, ##*P* < 0.01, ###*P* < 0.001 compared to the decellularized group; △*P* < 0.05, △△*P* < 0.01, △△△*P* < 0.001 compared to GA-crosslinking group; ▲*P* < 0.05, ▲▲*P* < 0.01, ▲▲▲*P* < 0.001 compared to 2 mg/ml Cor-group.

Then, to explore cell adherence, the cytoskeleton of ECs seeded on samples was observed by CLSM. As Figure 4A shows, cells on decellularized- and GA-crosslinked samples exhibited a small spherical shape, indicated non-adherent morphology, and failed to form monolayer protection. While ECs exhibited optimal attachment and spread on Cor-crosslinked tissues and even formed confluent monolayers for rapid re-endothelialization. An analogous phenomenon was also observed from SEM images (Figure 4B). ECs changed to polygon-like morphology after 4 days seeding on Cor-crosslinked samples, evenly, some cells tiled over the massive filopodia and formed a dense cell layer. The enhancement of cell adhesion and proliferation may be attributed to a large amount of hydroxyl groups in Cor. It possessed a similar chemical structure to epigallocatechin-3-gallate (EGCG), which has proved to lead to faster and better-organized endothelialization. Firstly, they can form strong H-bonds with soy proteins, providing cell-adhesion sites to enhance their interface contact with cells [36–38]. Then, polyphenol in Cor has a strong antioxidant effect, which can inhibit the endothelial damage caused by oxidative stress and avoid inflammatory factors coverage on blood vessels. Additionally, Cor-crosslinking enlarged the surface roughness of tissues, which is fit for cell adhesion and proliferation.

Moreover, the endothelialization process is related to some cytokines, which are expressed at EC–cell junctions and are very important in restoring endothelial integrity. Among them, vascular endothelial growth factor (VEGF) is effective in maintaining permeability and endothelial proliferation. Transforming growth factor-β (TGF-β) is regarded as the critical factor in accelerating angiogenesis and tissue regeneration [27, 39, 40]. As Figure 4C exhibited, ECs on Cor-crosslinked samples expressed strong VEGF and TGF-β to the supernatant, indicating they could fully keep the integrity of the EC monolayer. In addition, they are concentration-dependent: With Cor concentration increased, the secretion level of VEGF and TGF-β gradually increased, and the largest was obtained at 1 mg/ml and 2 mg/ml. That is, Cor in appropriate concentration could effectively promote TGF-β expression, which plays a critical role in stimulating EC migration and angiogenesis. TGF-β could also promote macrophages polarization from M1 to M2 phenotype, further stimulating the endogenous VEGF secretion and thus accelerating re-endothelialization [41, 42]. It indicated that the sustained release of Cor has the potential to accelerate the tissues’ transition from the inflammation phase to proliferation one at the vascular anastomosis.

### Macrophage polarization and anti-inflammatory property

**Figure 5.**
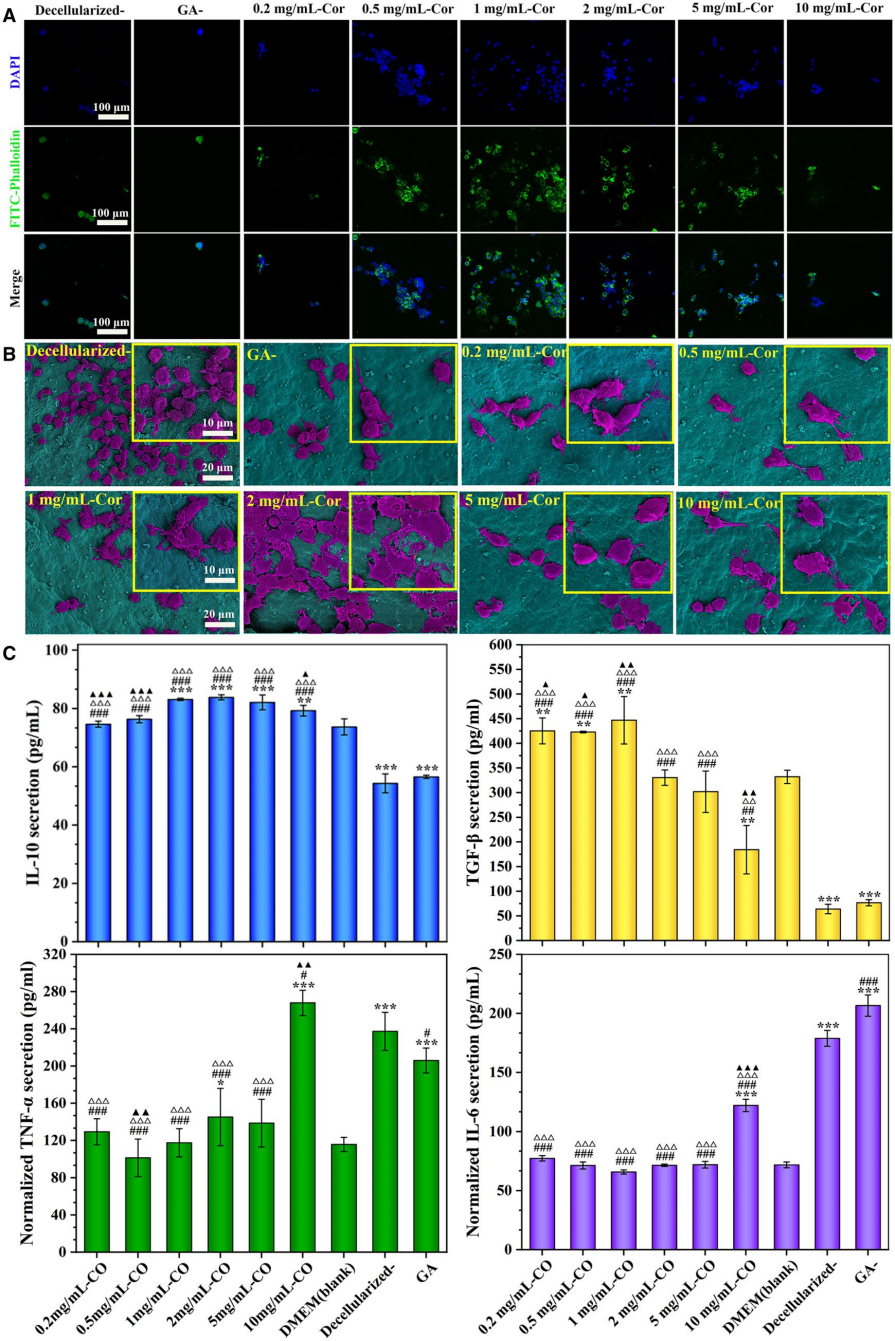
Analysis for macrophage polarization. (**A**) Cytoskeleton staining and (**B**) SEM observation of RAW 264.7 co-cultured with Cor-crosslinked tissues for 4 days. (**C**) Pro-inflammatory cytokines (TNF-α, IL-6) and anti-inflammatory cytokines (IL-10, TGF-β) secretion in supernatant. Among them, *P < 0.05, **P < 0.01, ***P < 0.001 compared to DMEM (blank) group; #*P* < 0.05, ##*P* < 0.01, ###*P* < 0.001 compared to the decellularized group; △*P* < 0.05, △△*P* < 0.01, △△△*P* < 0.001 compared to GA-crosslinking group; ▲*P* < 0.05, ▲▲*P* < 0.01, ▲▲▲*P* < 0.001 compared to 2 mg/ml Cor-group. All these data were obtained from at least three repeated experiments.

Local acute and chronic inflammations may lead to thrombosis, intimal hyperplasia, and even calcification of blood vessels. As reported, macrophages can polarize into pro-inflammatory (M1) or anti-inflammatory (M2) phenotypes in specific microenvironments. The ‘classically activated’ M1 phenotype usually promotes an inflammatory response, while the ‘alternatively activated’ M2 tends to participate in immune regulation and tissue regeneration [33, 43]. As exhibited in Figure 5A, the area fraction of macrophages on Cor-crosslinked tissues is up to 20%, and abundant F-action indicating high viability was visible. It implied infeasibility for Cor-crosslinked tissues to suppress inflammation via decreasing macrophage survival rate. Noteworthiest, macrophages on Cor-crosslinked tissues displayed fusiform and extended pseudopodia indicating M2 phenotype, while cells on decellularized- and GA-crosslinked samples are round or egg-like shape, which were regarded as characteristic morphology for M1 phenotype (Figure 5B). Therefore, Cor modification on tissue surface could effectively convert macrophages polarization from M1 to M2 phenotype and exhibited a potential to promote anti-inflammatory response at the vascular anastomosis.

ELISA measurement in Figure 5C showed that TNF-α and IL-6 (M1 makers) secretions were significantly downregulated after Cor-crosslinking, while M2 makers expressions such as IL-10 and TGF-β were relatively upregulated. Moreover, they exhibited a significant concentration dependence. With the concentration of Cor increased, M1 makers’ expression gradually decreased and reached the lowest level at 2 mg/ml Cor-concentration (TNF-α, IL-6). Then, the corroborative trend was also obtained in M2 makers’ secretion, and 2 mg/ml Cor was thus selected as the optimal crosslinking concentration. It indicated that pure decellularization may promote macrophages to pro-inflammatory M1 phenotype and produce a series of inflammatory cytokines. Their applications have a risk of causing endothelial dysfunction, tissue destruction, and even blood vessel calcification, while macrophages on Cor-crosslinked samples mainly displayed M2 phenotype and secreted high levels of anti-inflammatory cytokines [44, 45]. They not only inhibit chronic inflammations at vascular anastomosis but also stimulate EC proliferation to achieve rapid endothelialization and promote vessel regeneration.

### ROS analysis for inflammation and macrophage polarization

**Figure 6.**
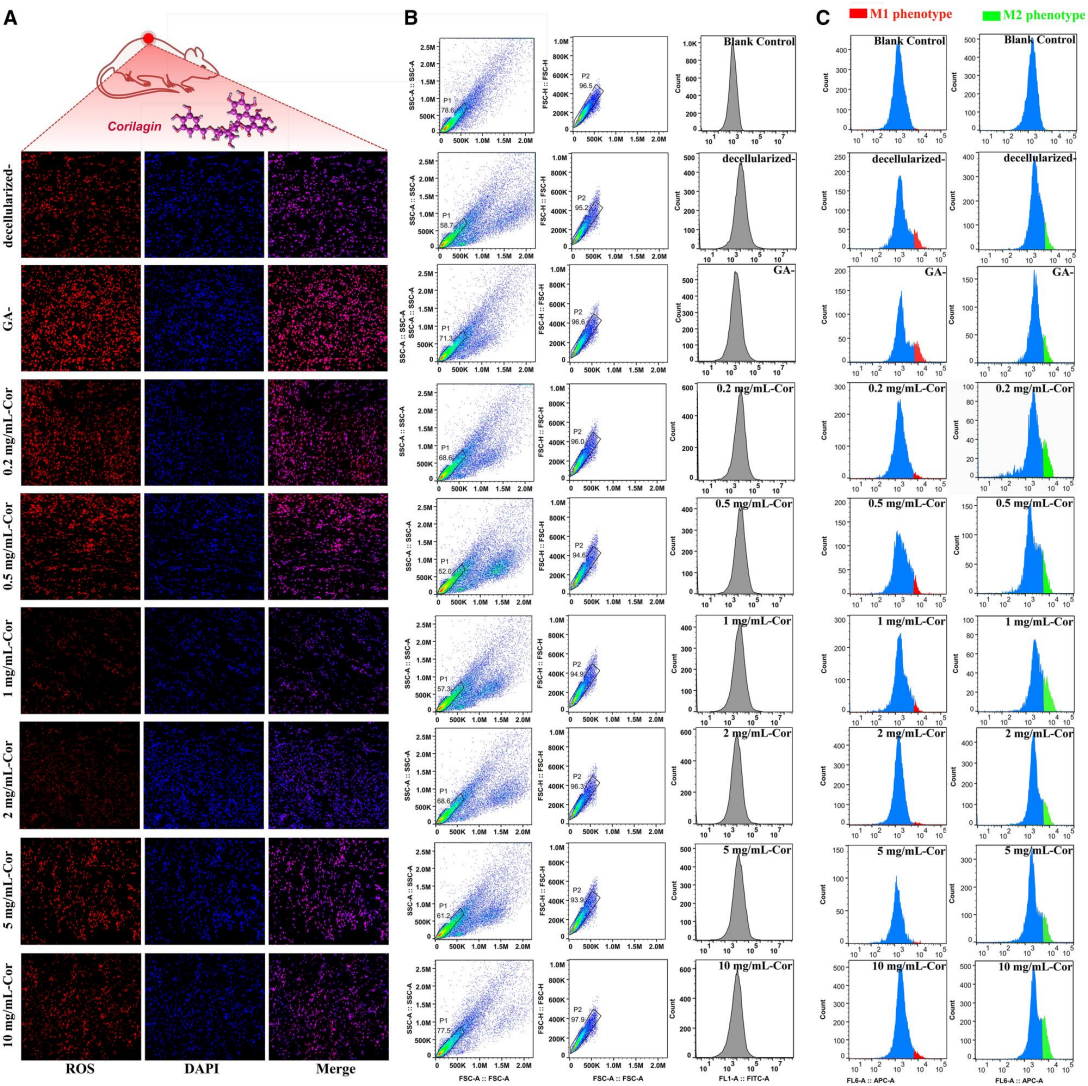
Cor crosslinking controlled ROS production in the implantation area. (**A**) Images of ROS distribution by fluorescence microscope. (**B**) FCA analysis of ROS production. (**C**) FCA analysis of CD86^+^ and CD206^+^ for macrophage polarization.

Generally, local inflammation and oxidative stress may increase the ROS produced at anastomosis. These excessive ROS would cause oxidative damage to various bioactive molecules and even accelerate the progression of many inflammatory-associated cardiovascular diseases, such as intimal hyperplasia, vascular stenosis, calcification and acute thrombus [46, 47]. Thus, ROS analysis was carried out, and the results are shown in Figure 6A and Supplementary Figure S9. Tissues adhered to decellularized samples and GA samples exhibited stronger red fluorescence than Cor groups, which indicated higher ROS levels and more serious inflammation risk. The quantitative analysis of ROS production by flow cytometry analysis in Figure 6B also demonstrated that Cor crosslinking could effectively scavenge ROS, and an obvious concentration dependence was shown: The ROS level decreased gradually with the increasing Cor concentration and reached down to the minimum value at 2 mg/ml, which is benefit to maintain ROS homeostasis and help alleviate oxidative stress.

Combined with the results in Figure 5, it was found that the reduction of ROS and the transformation of macrophages into M2 phenotype occurred simultaneously. It indicated that suppressed ROS production after Cor crosslinking can effectively inhibit pro-inflammatory signals, and this process is probably related to macrophage phenotype transformation [43]. That is, ROS consumption in Cor groups has the potential to promote macrophages polarization from M1 to M2. Generally, macrophages participate in various pathological processes. M1 tends to release pro-inflammatory cytokines and accelerate acute pro-inflammatory responses. But once M1 polarized to M2 phenotype, they began to down-regulate inflammation via secreting growth factors (VEGF and TGF-β) and support organization regeneration. Reversely, the pro-inflammatory M1 phenotype could induce high ROS expression that will intensify inflammatory actions and affect inflammation factors secretion, resulting in thrombosis or accelerating it. As exhibited in Figure 6C, the macrophage polarization to regenerative M2 phenotype can be greatly promoted in the presence of Cor in appropriate concentration. With Cor concentration increasing from 0.25 mg/ml to 2 mg/ml, the transformation rate of CD86^+^ decreased from 14.71% to 1.87%, while that of CD206^+^ increased from 2.94% to 17.16%. This indicated successful regulation of inflammatory stimulation in initial implantation, which is regarded as the most critical link to maintaining long-term vascular patency.

### *In vivo* histocompatibility and re-endothelialization

Excessive ROS increases the risk of endothelial permeability, and these leakages may further destroy the integrity of the endothelial layer. Rapid re-endothelialization is critical to maintaining vessels’ long-term patency via forming a tight-knit and confluent endothelial monolayer as a semipermeable barrier. It could effectively prevent blood contact, thus avoiding platelet aggregation and acute thrombus [4, 33, 35].

**Figure 7.**
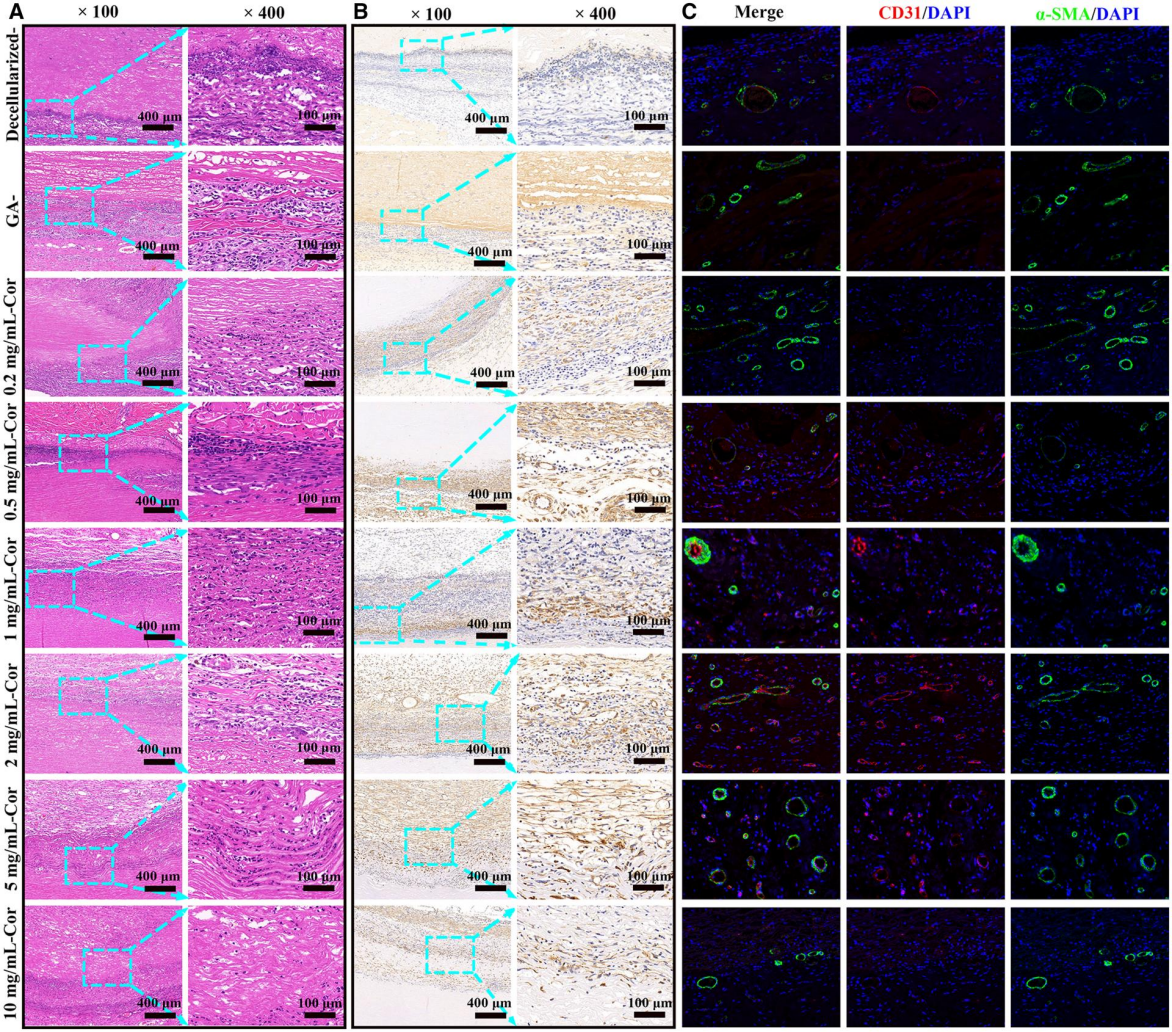
*In vivo* vascular generation after 4 weeks implantation. (**A**) H&E staining for histocompatibility analysis. (**B**) Immunofluorescence staining of CD31 and α-SMA for blood vessel co-localization. (**C**) IHC with VEGF antibody for quantitative analysis of vascular function.

As exhibited in H&E stains (Figure 7A), a more intact monolayer was visible on Cor-crosslinked groups, especially when there are large numbers of ECs arranged in a ring in 2 mg/ml-Cor group, and these regularly distributed cells are regarded as the initial state of angiogenesis. It indicated that Cor in appropriate concentration has the potential to accelerate endothelial proliferation. Other studies also suggested that polyphenols could easily bind to tissues and form rich hydrogen bonds crosslinking. It was helpful to promote angiogenesis-related factors secretion and further accelerate vascular repair [5, 16]. Nevertheless, only sporadic normal cells and large numbers of inflammatory cells were visible around GA- and decellularized-control, probably leading to acute thrombosis and advanced calcification. In addition, although the number of inflammatory cells reduced in low-concentration-Cor groups (0.25 mg/ml and 0.5 mg/ml), tissue cavities and sparse fiber distribution were still obvious, which indicated incomplete crosslinking and early degradation, even increasing the risk of aneurysm after vascular implantation. It also should be noticed that there is still a risk of possible incompatibilities of xeno-genic tissue for human implants because of some xeno-antigens (such as alpha Gal and Neu5Gc) are widely present in animals but not in human.

Then, ECs’ recruitment and coverage rate on blood vessels were further detected by immunohistochemistry and immunofluorescent staining. As exhibited in Figure 7B, CD31 and α-SMA co-localization were employed to characterize microvascular generation. Among them, the 2 mg/ml-Cor group was almost fully covered by cobblestone-like ECs, providing a denser protective layer and higher bioactivity. Furthermore, ECs arrangement was homonymous and along the tangent direction of vessel diameter, which indicated better endothelialized functions. They tend to align along the bloodstream rapidly and form two-layered co-localization with adjacent red and green fluorescence, pretty similar to native blood vessels. Evenly, some areas in 2 mg/ml Cor samples only exhibited CD31 signals, which indicated ECs maintained a competitive growth advantage over SMCs, and it is hopeful of maintaining long-term patency and avoiding intimal hyperplasia [22]. Nonetheless, some abnormal blood vessels in decellularized- and GA-groups exhibited α-SMA positive signals only, they lack monolayers’ protection, and vascular restenosis occurred more easily.

Immunohistochemistry (IHC) analysis was further employed to quantitatively evaluate vascular function. As seen in Figure 7C, a strongly positive expression of VEGF (the most critical endothelium-related factor) was visible in the 2 mg/ml Cor-treated group, indicating rapid vascular reconstruction and active vascular function. That may be explained as follows: Firstly, Cor is a kind of active water-soluble polyphenol that could be extracted from plants of Euphorbiaceae Phyllanthus, exhibiting perfect biocompatibility and bioactivity. For traditional crosslinking reagents, some functional groups will react with cellular protein directly, resulting in changes in their permeability and even a decrease in their proliferative activity [13, 15]. Fortunately, Cor could provide stable cross-linkages via abundant hydrogen bonds without introducing other unnecessary functional groups to avoid the risk of cell degeneration. Then, as a recognized antioxidant, Cor could effectively remove ROS at implanted areas and avoid endothelial damage caused by oxidative stress [14, 17]. In addition, ROS consumption could also regulate VSMC’s phenotype transformation and reduce the intimal hyperplasia caused by VSMC’s excessive proliferation and abnormal migration. Thus, large amounts of active phenolic hydroxyls in Cor could induce rapid endothelialization in the initial period, exhibiting a potential to prevent early thrombosis and maintain long-term patency.

### *In vivo* inflammatory resolution

**Figure 8.**
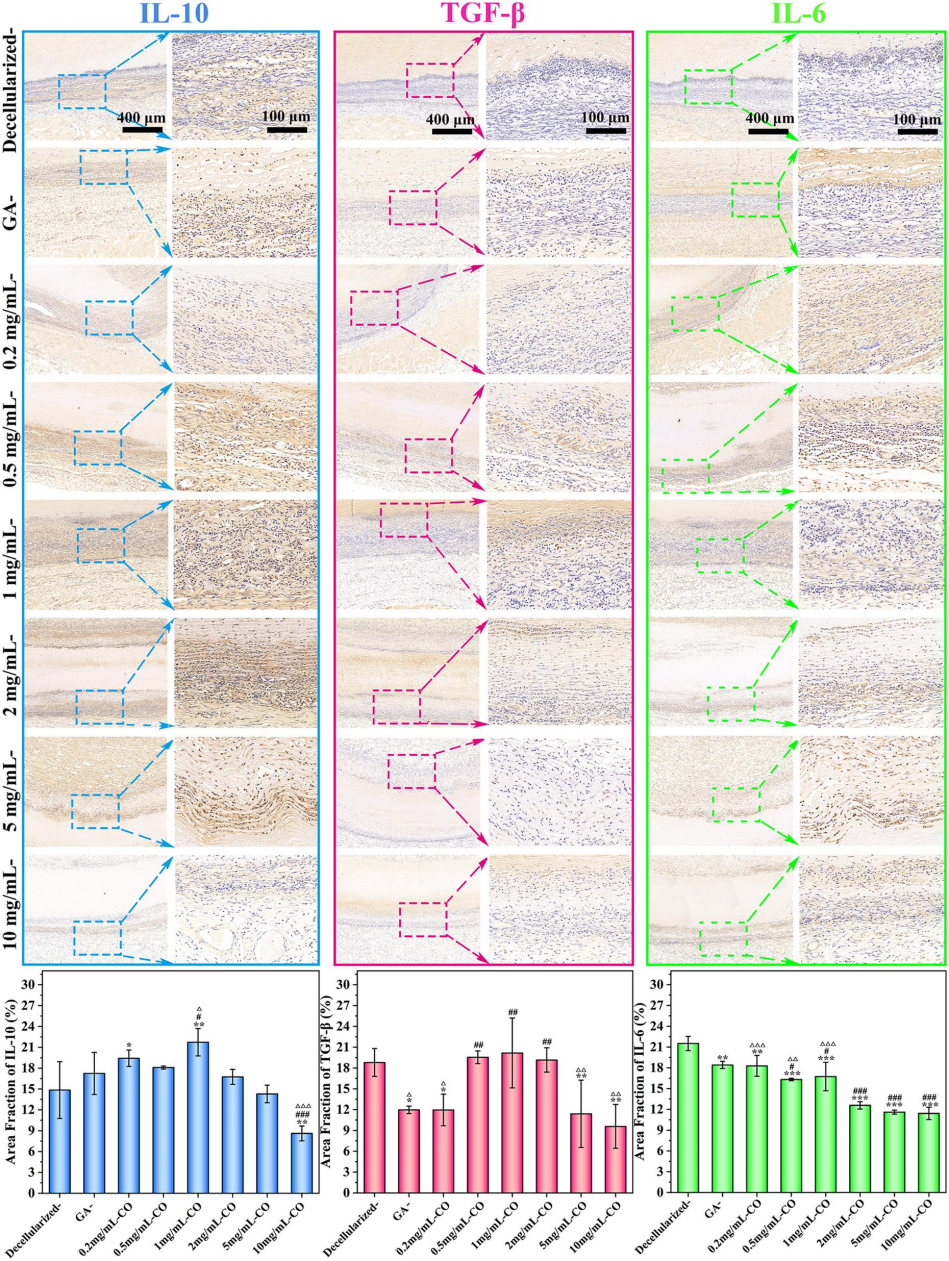
IHC analysis for the inflammatory response *in vivo* with antibody to pro-inflammatory (IL-6) and anti-inflammatory (TGF-β and IL-10) factors expression after 4-week subcutaneous implantation. Among them, *P < 0.05, **P < 0.01, ***P < 0.001 compared to decellularized group; #*P* < 0.05, ##*P* < 0.01, ###*P* < 0.001 compared to GA-crosslinking group; △*P* < 0.05, △△*P* < 0.01, △△△*P* < 0.001 compared to 2 mg/ml Cor-group. All data in the bar graphs were obtained from at least three repeated experiments.

Persistent inflammation has a risk of inducing collagen damage, acute thrombosis, intimal hyperplasia and calcification, even finally causing failure of blood vessel implantation. During this period, excessive ROS production is regarded as the critical triggering factor to oxidative stress *in vivo*, promoting the transformation of VSMC phenotype from contractile to synthetic one and accelerating inflammatory cytokines (TNF-α, IL-6) secretion [33, 43]. As known, TNF-α and IL-6 are potent pleiotropic cytokines that participate in various pathological reactions for inflammatory properties. In Figure 8 and Supplementary Figure S10, strong positive staining of them was exhibited in GA- and decellularized- groups, indicating severe inflammation. Slightly different, inflammatory cells infiltrated and accumulated toward GA samples while just migrated to the degraded cavity in the decellularized group. Then, samples crosslinked by Cor showed lower expression of pro-inflammatory cytokines, and the minimum value was obtained at 2 mg/ml point. Whereas anti-inflammatory factors of TGF-β and IL-10 were significantly upregulated in Cor groups, suggesting enhanced resolution of inflammation and better endothelialization. IL-10 is critical to inhibit the activation of inflammatory cells, and TGF-β could inhibit ECM degradation and avoid macrophage recognition. It indicated that their well-preserved fibers could reduce pro-inflammatory responses caused by unstable degradation products at anastomosis. In addition, ROS consumption achieved by polyphenols could inhibit the transformation of VSMC to the pro-inflammatory phenotype and further inhibit the release of inflammatory-related factors, such as IL-6 and TNF-α, from synthetic VSMC [5, 39]. This process exhibited concentration dependence, and the 2 mg/ml Cor group is superior to any others: it can suppress inflammation rapidly and recruit the fewest positive cells to target anastomotic stoma.

Briefly, it is very critical to control postoperative inflammation to avoid it participating in pathological processes, thus preventing thrombosis and intimal hyperplasia in vascular repair [24]. Cor introduction effectively consumed ROS and controlled the inflammatory response, which provided a theoretical basis for regulating the inflammatory cycle.

**Figure 9.**
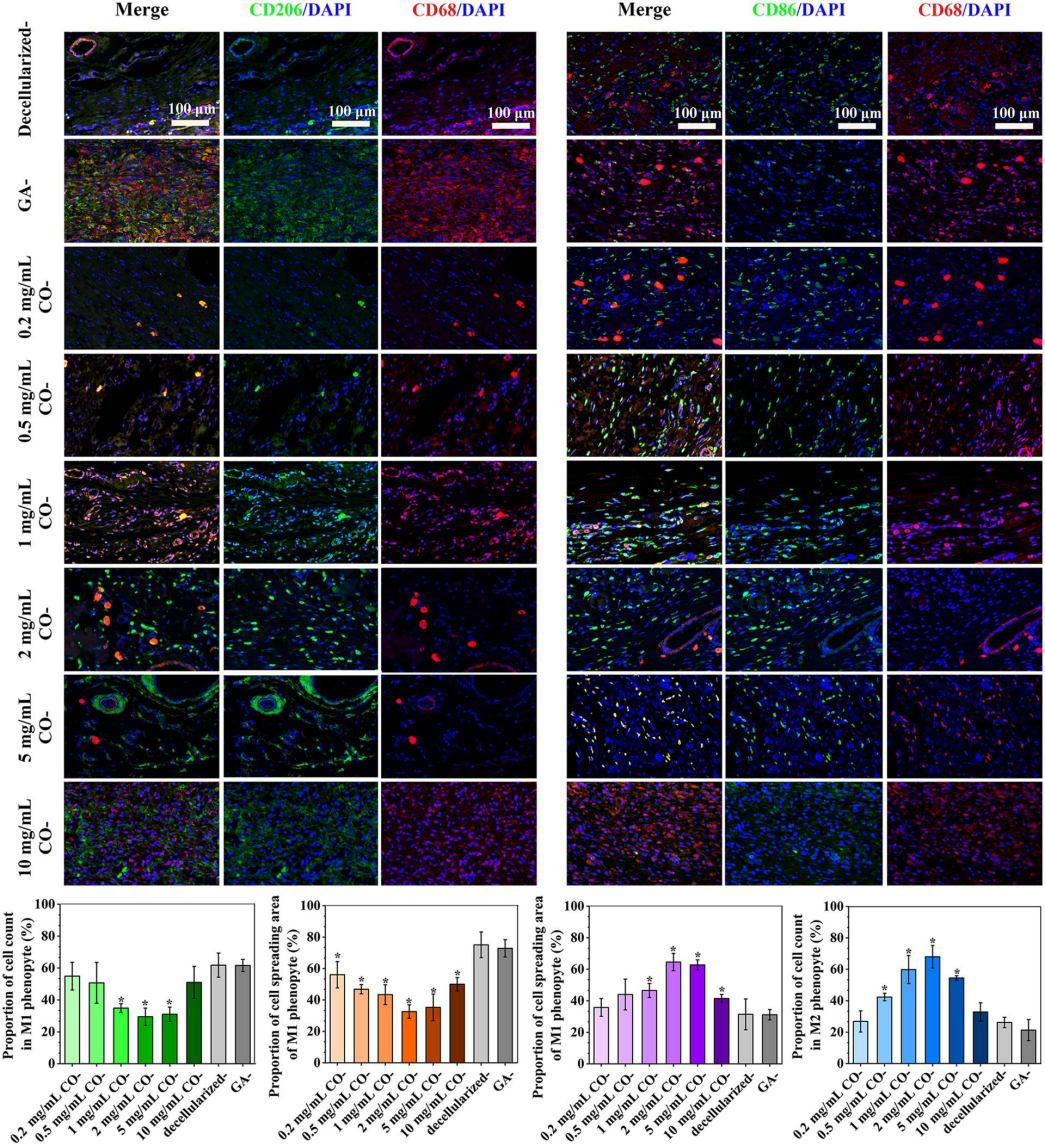
Immunofluorescence staining of infiltrating macrophages, which labeled pan-macrophage phenotype with CD68, M1 phenotype with CD86 markers and M2 with CD206 after 4-weeks implantation (* means significant difference compared to decellularized sample, *P < 0.05). All data in the bar graphs were obtained from at least three repeated experiments.

As previously reported, inappropriate macrophage activation may exacerbate inflammation and destroy healthy tissue/implant integration. CD86 macrophage induces high expression of pro-inflammatory cytokines, whereas CD206 ones prefer to secrete anti-inflammatory cytokines. As exhibited in Figure 9, there was a large number of macrophages signaled CD86 and sporadic ones signaled CD206 around decellularized- and GA-crosslinked samples, which indicated their toxic leachates or degradation products might cause strong and long-term foreign body reactions, and this process is favorable for M1 polarization. Correspondingly, large numbers of CD206 signals were visible on Cor-crosslinked samples. This implied that their inflammation was effectively inhibited and Cor could promote macrophages polarization from M1 to M2 phenotype, thus promoting anti-inflammatory factors secretion. Similar results were also confirmed by SEM observation and ELISA measurement (Figure 5) *in vitro*. We suspect that Cor crosslinking may accelerate ROS consumption, thereby altering macrophage polarization from the M1 phenotype to the M2 one. It can also enhance the expression of VEGF, TGF-β and IL-10, which are key regulators of endothelialization and anti-inflammation that will synergistically enhance tissue regeneration.

### *In vivo* anti-calcification

**Figure 10.**
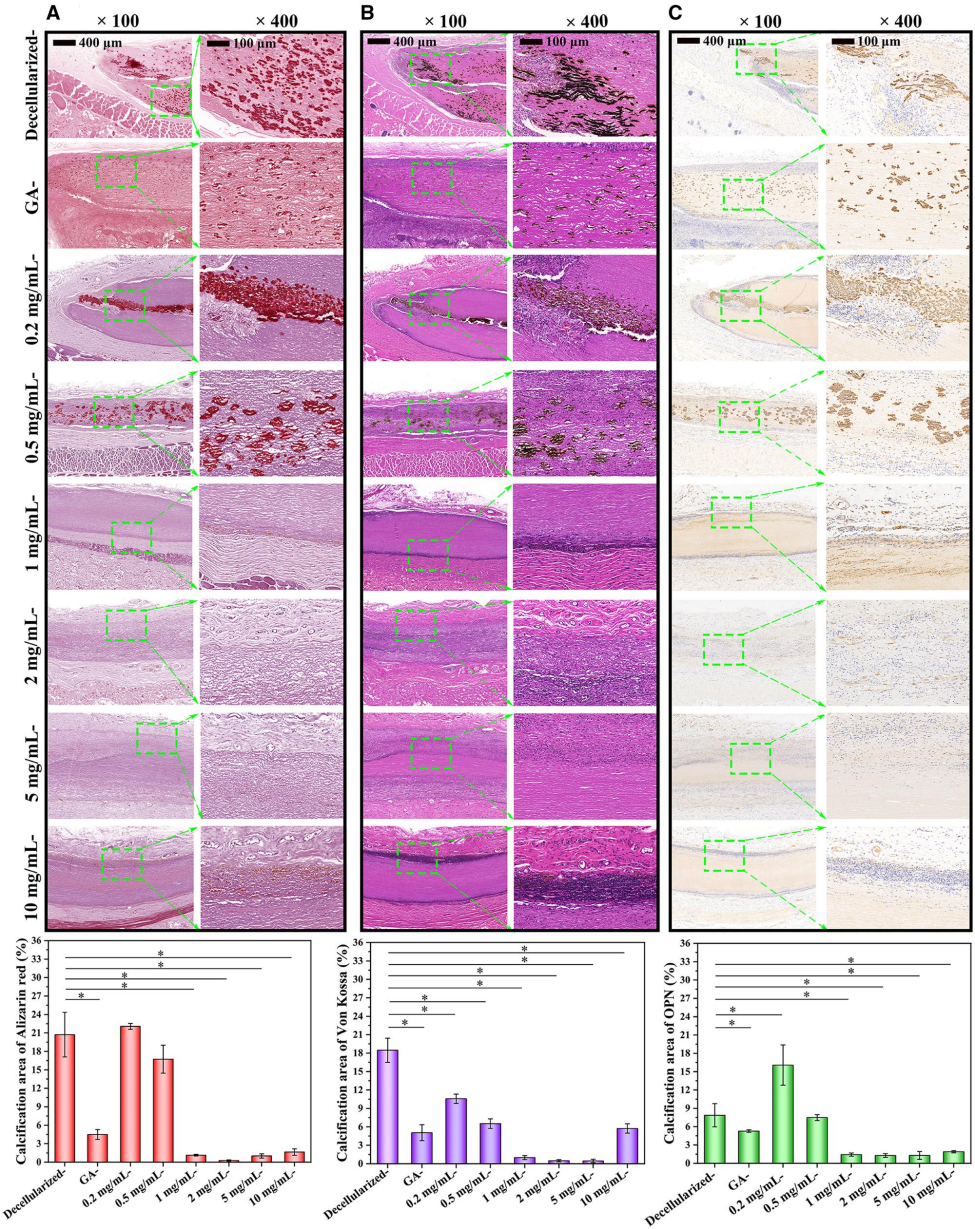
Image of mineral deposition stained with (**A**) Von Kossa, (**B**) Alizarin red and (**C**) OPN after 12-weeks subcutaneous implantation in SD rats (* means significant difference compared to decellularized sample, *P < 0.05). All data in the bar graphs were obtained from at least three repeated experiments.

Generally, the large amount of released ROS can promote endothelial damage, which will accelerate the development of chronic inflammation, thereby activating calcification-related genes and causing vascular calcification. Calcification has emerged as another important inducement for occlusion and may lead to vessels’ implantation failure. It tends to aggravate vascular stenosis and finally develops into thrombosis or blocking [12, 35]. As exhibited in Figure 10A, Cor-treated ones showed few calcification deposits, while decellularized- and GA-crosslinked control manifested many more after 12 weeks of implantation. Especially there are several voids and many fiber-loose regions in decellularized groups, and calcification just occurred in these areas or tightly surrounded damaged fibers. That may be because various toxic substances originated from degradation products, and residual immunogenicity will act as calcification sites locally, and they prefer to accelerate massive mineral depositions formed as well. Besides, decellularized tissues are rich in free amino acids. They expose anionic groups such as -COOH, -SH and -OH, which exhibited strong electrostatic force to Ca^2+^ and easy-to-form nucleation sites for HA minerals. Although GA-fixation could effectively prevent degraded cavities, tissues are still predisposed to calcification of their high-strength binding to Ca^2+^. Moreover, GA fixation was ineffective in stabilizing elastin, and their disruption/entanglement since enzymolysis attack was another critical factor in causing serious calcification. In contrast, the deposition of minerals on Cor-crosslinked groups was significantly inhibited depending on Cor concentration. After Cor treatment in appropriate concentration (2 mg/ml), only sporadic small apatite deposits were visible on fibers, showing their mineral nucleation and deposition had reached the lowest value. It was also accompanied by the distinctively decreased signal intensity in Alizarin red staining in Figure 10C. That may be because Cor formed dense H-bonds to tissues and acted as stable barriers to maintain the integrity of elastin without calcification, and the most suitable crosslinking concentration is displayed at 2 mg/ml. Then, with the concentration of Cor higher or lower, their calcified inhibition was weakened. Evenly, there was visible calcium accumulation in 0.2 mg/ml- and 10 mg/ml-groups.

Similar results of OCN and OPN staining were further obtained in Figure 10C and Supplementary Figure S11. It showed that mineral depositions on decellularized- and GA-crosslinked samples were mature and large, while calcification spots on various Cor groups were initial and almost imperceptible.

## Conclusion

In summary, we developed a novel Cor crosslinking on biological blood vessels to accelerate endothelialization and suppress inflammation at the vascular anastomosis. It can form a stable network to resist enzymatic hydrolysis via H-bonds but not introduce other toxic functional groups, which was confirmed by ^1^H NMR analysis and biomechanical test. Cor crosslinking simultaneously endowed tissues with multifunctional bioactivities, including anti-inflammatory and anti-oxidation. *In vivo* and *in vitro* tests confirmed that Cor could consume ROS to exert anti-inflammatory effects and establish a favorable microenvironment at the vascular anastomosis. It was proved that Cor could improve ECs adhesion, promote their rapid coverage on tissues, and maintain optimal hemocompatibility. Meanwhile, Cor promoted macrophages polarization from M1 to M2 phenotype, which is the key regulator to induce inflammation resolution and enhance tissue regeneration via promoting anti-inflammatory cytokines (IL-10 and TGF-β) secretion. In addition, the anti-calcification property was elucidated by subcutaneous implantation. Finally, the multiple functions of Cor exhibited a significant concentration dependence, and 2 mg/ml was selected as the optimal concentration for future clinical application. Therefore, our study has the potential to provide a novel method to maintain the long-term patency of artificial small-diameter blood vessels by avoiding acute thrombosis, serious inflammation and chronic calcification.

## Supplementary Material

rbae074_Supplementary_Data

## Data Availability

All data are available in the main text or the supplementary materials.
