# Supplementary material for: Corilagin functionalized decellularized extracellular matrix as artificial blood vessels with improved endothelialization and anti-inflammation by reactive oxygen species scavenging
Source: Regen Biomater. 2024 Jul 1;11:rbae074. doi: 10.1093/rb/rbae074 (PMC11631697; doi:10.1093/rb/rbae074)
Supplement: rbae074_Supplementary_Data [file rbae074_supplementary_data.docx]

**Supplementary Information**

**Corilagin functionalized** **decellularized extracellular matrix as artificial blood vessels with improved endothelialization and anti-inflammation by** **reactive oxygen species scavenging**

Xu Wang,^a^ * Hanmei Fu,^a^ Huibin Wu,^b^ Xiaohua Peng,^a^ Xu Peng,^c^ Xixun Yu^c^, Hui Liu^d^, Junmei Wu,^a,e^* Ling Luo,^a^ Shan Yan,^a^ Xinglin Cheng,^a^ Xiong Zhou,^f^ * Xiangyang Yuan^b^ *

a. School of Acupuncture and Tuina, Chengdu University of Traditional Chinese Medicine, Chengdu 610075, China

b. College of Science, Sichuan Agricultural University, Ya’an 625014, China

c. College of Polymer Science and Engineering and Laboratory Animal Center, Sichuan University, Chengdu 610065, China

d. School of Intelligent Medicine, Chengdu University of Traditional Chinese Medicine, Chengdu 610075, China

e. College of Traditional Chinese Medicine, The University of Hong Kong, Hong Kong, 999077, China

f. Department of Biomedical Engineering, City University of Hong Kong, Hong Kong 999077, China

*Corresponding author: E-mail address: yxyang1990@163.com, Tel: +86-28-87683396 (Xu Wang), Present address: School of Acupuncture and Tuina, Chengdu University of Traditional Chinese Medicine, Chengdu 610075, China.

*Co-corresponding author: E-mail address: xyyuan@sicau.edu.cn, Tel: +86-0835-2886189. (Xiangyang Yuan), Present address: College of Science, Sichuan Agricultural University, No.46, Xin Kang Road, Ya’an, Sichuan Province, 625014, China.

*Co-corresponding author: E-mail address: xiongzhou2-c@my.cityu.edu.hk, Tel: +86 18231351197 (Xiong Zhou), Present address: Department of Biomedical Engineering, City University of Hong Kong, Hong Kong, 999077, China.

*Co-corresponding author: E-mail address: wujunmei@126.com, Present address 1: School of Acupuncture and Tuina, Chengdu University of Traditional Chinese Medicine, Chengdu 610075, China. Present address 2: College of Traditional Chinese Medicine, The University of Hong Kong, Hong Kong, 999077, China.

**Table of Contents**

1. Supplementary Experimental methods 3

1.1 Materials and reagents 3

1.2 Fixation index (FI) determination 3

1.3 Weight loss detection in enzymolysis 3

1.4 Cell culture and Seeding 4

1.5 The operational process of animal experiments 4

1.6 The accumulated corilagin release detected by UV absorption 4

1.7 Chemical composition of fluid for tissues trypsin digestion 5

1.8 Coagulation and whole blood incubation 5

2. Supplementary Results 6

2.1 Mechanical properties of corilagin crosslinked tissues 6

2.2 Mechanical strength of corilagin-crosslinked samples after SBF immersion 6

2.3 Corilagin solution in gradient concentration after crosslinking 7

2.4 Fixation index (FI) measurement 8

2.5 Relative weight loss after collagenase and elastase digestion 9

2.6 Corilagin releasing 10

2.7 Plasma coagulation parameters in vitro 11

2.8 Complement activation in vitro 12

2.9 Hemolysis rate (HR) analysis 13

2.10 Cell viability of HUVECs 16

2.11 ROS analysis for inflammatory risk 17

2.12 IHC analysis for inflammatory response in vivo 18

2.13 In vivo mineral deposition 19

**1.** **Supplementary Experimental methods**

**1.1 Materials and reagents**

All chemicals were of biological/analytical reagent grade and could be directly used without further purification. Among them, Corilagin (Cor-, purity＞98%) was purchased from Herbpurify CO., LTD (Chengdu, China). Dimethyl sulfoxide (DMSO) and glutaraldehyde (GA) were obtained from Aladdin Industrial Corporation (Shanghai, China). EDTA and Triton X-100 were purchased from Amresco Co. (CLE, USA), while, streptomycin, penicillin, trypsin, fetal bovine serum (FBS) and Dulbecco’s modified Eagle’s medium (DMEM) were supplied by Gibco (Grandlsland, NY, USA). The HUVECs were obtained from West China Hospital, Sichuan University (China).

**1.2 Fixation index (FI) determination**

As a sensitive amino indicator, ninhydrin (NHN) could easily react with free amino groups on decellularized tissues through boiling to form a blue-purple water-soluble substance. Its optical absorbance was directly proportional to the amount of unreacted free amino groups in tissues. Therefore, FI was measured using NHN assay to quantitatively evaluate the crosslinking degree. Briefly, after being fixed for designated periods (15min to 72h), tissues were promptly taken out and lyophilized for 24h. Subsequently, these lyophilized tissues were boiled in NHN solution for 20 min and the OD value of solution was measured by spectrophotometer (UV-752, Shanghai) at 570 nm. A standard curve recording the measured value of various known concentrations of glycine was plotted, and the FI was calculated according to the following formula:

$$\mathrm{FI}\left( \% \right)=\frac{{{(NHN}_{reactive amine})}_{decellularized}-{({NHN}_{reactive amine})}_{crosslinked}}{{NHN}_{reactive amine}}$$

**1.3 Weight loss detection in enzymolysis**

Since natural tissues are mainly composed of collagen and elastin, two kinds of related enzymes are employed in hydrolytic assessment for weight loss detection. In detail, the enzymolysis solution was processed in Tris buffer (0.1M, pH=7.4) containing collagenase Ⅰ (125 U/mL) and elastase (30 U/mL). Then, the lyophilized specimens were weighed (W_0_) and immersed in enzymatic mixture solution for predetermined period (0.5 h, 1 h, 3 h, 6 h, 12 h and 24 h). Followed by terminated hydrolytic process via adding 10 mM EDTA solution at every interval point, the undigested parts were lyophilized again and weighted as W_t_. The percentage of weight loss (ΔW%) was calculated as follows:

$$\Delta W\%=\frac{W_{0}-W_{t}}{W_{0}}\times100\%$$

where W_0_ represents samples’ initial weight and W_t_ represents the final value of corresponding one after enzymatic hydrolysis.

**1.4 Cell culture and Seeding**

***Cell culture and seeding****.* Human umbilical vein endothelia cells (HUVECs, passage 3-5) purchased from West China Hospital were maintained in complete cell medium supplemented with 10% fetal bovine serum (FBS, BI, Israel), 1% penicillin-streptomycin (Gibco) at 37^o^C in an atmosphere of 5% CO_2_ and 95% relative humidity for cell proliferation and morphology detection. Prior to cell seeding, samples were lyophilized and sterilized via gamma irradiation (^60^Co γ-rays, 25 kGy). Then, HUVECs were digested and seeded on various samples at a density of 2×10^4^ cells/well. Each medium was refreshed every 2 days.

***Cell proliferation****.* After 1-, 3-, 5- and 7-days incubation, wells were rinsed with PBS for several times and all samples were carefully transferred into a new 24-well plate. Soon after that, fresh cell medium with 200 μL of CCK-8 working solution was added and co-cultured for 1 h. After their soluble orange crystals are uniform, cell proliferation of each sample could be correspondingly measured through reading their absorbance value at 490 nm with a micro-plate reader.

**1.5 The operational process of animal experiments**

Prior to implantation, lyophilized tissue samples were trimmed to 1 cm × 1 cm squares and sterilized by ^60^Co irradiation. Six parallel samples were guaranteed for each experimental group. After 108 female SD rats (6 weeks in age and 150 g in weight) were anesthetized with isoflurane gas inhalation, two symmetrical and longitudinal surgical incisions were made on either side of the back of each rat. The modified samples were immersed in sterile saline to restore softness, and soon afterwards they were implanted into subcutaneous pockets. Their incisions were carefully closed with 4-0 surgical staples. After scheduled implantation period (4-, 8-, and 12-weeks), samples containing surrounded newly formed capsule were harvested and fixed with formalin overnight, and they were applied to further evaluation of the revascularization, anti-inflammatory and anti-calcification.

**1.6 The accumulated corilagin release detected by UV absorption**

Before corilagin release measurement, all crosslinked tissues were lyophilized and weighed. Then, we immersed samples into simulated body fluids (SBF) to simulate the internal environment, and the accumulated concentration of corilagin was detected via UV absorption (269 nm) at pre-determined periods (1h, 2h, 4h, 8h, 12h, 24h, 36h, 48h, 72h, and 96h, respectively). Their OD values were recorded, which are proportional to the concentration of corilagin in solution, and employed to estimate its release.

**1.7 Chemical composition of fluid for tissues trypsin digestion**

Except for preserving intact fibers microstructure, some bioactive factors, which could effectively promote blood vessels repair and accelerate their fusion to surrounded tissues, were also needed to maintain in original tissues. It was critical to maintain blood vessels long-term patency. Therefore, a mild condition that could ensure complete decellularization without damaging active factors will be picked out. In our research, trypsin was added for cells digestion: it could prevent cells adhering to tissues surface and accelerate cells dropping.

**Table S1.** Chemical composition of fluid for tissues trypsin digestion

|  | **Solution Composition** | **Component Content** |
| --- | --- | --- |
|  | trypsin (Sigma) | 0.25% |
| **Trypsin digestion fluid** | EDTA solution (Gibco) | 0.02% |
|  | PBS solution (Hyclone) | 0.1 M, pH 7.2 |

**1.8 Coagulation and whole blood incubation**

The whole blood was obtained original from cardiac blood collection. After local disinfection, the point with the strongest heartbeat was find out by fingers touch, and a needle was inserted to draw blood out. The obtained whole blood was preserved in anticoagulant tube immediately (anticoagulants: 2.5% sodium citrate, and the ratio of anticoagulant to blood is 1:10).

The whole blood incubation assay was described as follow: The anticoagulant tube was slowly shaken and maintained in ice box. Meanwhile, all tissue samples were placed into 24 well-plate, and pre-immersed with physiological saline at 37 °C for 1 hours. Then, their solution were discarded, and 1 mL of the whole blood was replaced to each sample and maintain immersion at 37 ℃ for 1 hours. After that, the whole blood was taken out and centrifuged at 2500 rpm for 4 h to obtain plasma for further ELISA treatment. Besides, the immersed tissue samples were rinsed with sterile PBS buffer for several times, and the attached cells from blood were fixed by 2.5% GA for SEM observation.

**2. Supplementary Results**

**2.1 Mechanical properties of corilagin crosslinked tissues**

In order to serve as artificial blood vessels with optimal functions during implantation, adequate mechanical strength is required for fixed tissues. As shown in **Table S2**, corilagin-modification significantly increased the mechanical strength of decellularized tissues. The value of “Ultimate tensile stress” and “Young’s modulus” for GA-fixed and corilagin-fixed tissues were significantly higher than that of the decellularized ones, while the values of “Ultimate tensile strain” decreased, which suggested decellularized tissues have better elasticity. Therefore, the mechanical strength of tissues increased when treated with crosslinking reagents. GA and corilagin have comparable crosslinking effect. With the concentration of corilagin increased, the ultimate tensile stress of crosslinked samples increased and achieved maximum of 1.542 ± 0.591 MPa at 1 mg/mL, which was exactly in accordance with the result of FI measurement. Furthermore, the Young’s modulus of corilagin-crosslinked tissues was also concentration-dependence, which also confirmed that effective crosslinking formed via numerous H-bonds introducing.

**Table S2.** Mechanical properties of various concentrations of corilagin crosslinked tissues. All data were calculated from at least 3 repeated experiments.

| **Treated samples** | **Ultimate tensile strain (%)** | **Ultimate tensile stress (MPa)** | **Young’s** **modulus** |
| --- | --- | --- | --- |
| Decellularized- | 84.023 ± 7.129 | 0.907 ± 0.128 | 1.795 ± 0.205 |
| 0.2 mg/mL Cor- | 62.689 ± 5.814 | 1.231 ± 0.197 | 2.790 ± 0.212 |
| 0.5 mg/mL Cor- | 96.114 ± 11.125 | 1.506 ± 0.071 | 2.845 ± 0.460 |
| 1 mg/mL Cor- | 79.999 ± 5.332 | 1.542 ± 0.591 | 3.055 ± 0.332 |
| 2 mg/mL Cor- | 76.242 ± 4.546 | 1.472 ± 0.478 | 2.920 ± 0.311 |
| 5 mg/mL Cor- | 71.698 ± 6.246 | 1.241 ± 0.232 | 3.100 ± 0.240 |
| 10 mg/mL Cor- | 86.699 ± 3.701 | 1.380 ± 0.205 | 2.925 ± 1.054 |
| GA- | 104.601 ± 20.777 | 2.485 ± 0.077 | 3.490 ± 0.792 |

**2.2 Mechanical strength of corilagin-crosslinked samples after SBF immersion**

We detected the biomechanical strength of samples after longer immersion to evaluate their structural stability. The left image represents the mechanical strength of tissues in each group that have not been immersed in SBF solution, while the right one reflects their strength value after 21 days immersion in SBF. As exhibited below, the biological tissues crosslinked by corilagin still maintain high mechanical strength after 21 days immersion in simulated body fluids (SBF). For each specific concentration, there are no significant differences in their mechanical strength before and after SBF soaking. It implied that corilagin formed multiple H-bonds to tissues and could act as a stable barrier to maintain high mechanical strength in a long period after implantation. In addition, the samples suffered from SBF immersion have an obvious yield stage. It implied that a small part of crosslinking bonds were released after long-term immersion, which induce their relative slip between long polymer chains.

**Table S3.** Mechanical strength of corilagin-crosslinked samples after 21 days SBF immersion. All datas were calculated from at least 3 repeated experiments.

| **Treated samples** | **Ultimate tensile strain (%)** | **Ultimate tensile stress (MPa)** | **Young’s modulus** |
| --- | --- | --- | --- |
| Decellularized- | 91.460 ± 6.600 | 0.875 ± 0.144 | 1.650 ± 0.143 |
| 0.2 mg/mL Cor- | 79.068±7.877 | 1.317 ± 0.099 | 2.583 ± 0.172 |
| 0.5 mg/mL Cor- | 82.947 ± 14.206 | 1.481 ± 0.140 | 2.317 ± 0.279 |
| 1 mg/mL Cor- | 77.886 ± 7.589 | 1.516 ± 0.159 | 2.575 ± 0.207 |
| 2 mg/mL Cor- | 73.040 ± 10.326 | 1.551 ± 0.209 | 2.378 ± 0.411 |
| 5 mg/mL Cor- | 79.995 ± 5.958 | 1.595 ± 0.133 | 2.547 ± 0.248 |
| 10 mg/mL Cor- | 76.6445 ± 2.319 | 1.581 ± 0.052 | 2.641 ± 0.375 |
| GA- | 78.178 ± 8.584 | 2.575 ± 0.373 | 3.150 ± 0.188 |

**2.3 Corilagin solution in gradient concentration after crosslinking**

Generally, the chemical structure of polyphenols is unstable. As well known, oxidation from polyphenols to quinones is one of the most important manifestations to exhibit their biological activity. After oxidation to quinones, the compounds have stronger antioxidant and anti-inflammatory properties, meanwhile, their solution will also darken in color. As exhibited in **Fig. S1**, with corilagin concentration increasing, the color of solution changed gradually from dark yellow to black. It indicated that there are more active quinone bonds in higher concentration of corilagin, which was fit for tissues crosslinking.

**
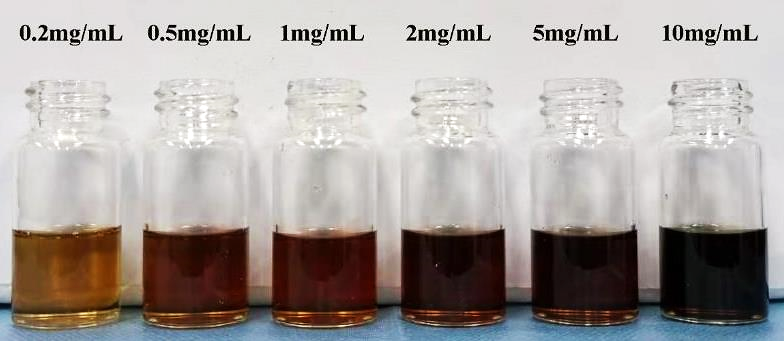
**

**Fig. S1.** Digital image of corilagin solution in gradient concentration after 72 h tissues crosslinking. Samples are crosslinked by corilagin solution with a concentration of 0.2, 0.5, 1, 2, 5 and 10 mg/mL

**2.4 Fixation index (FI) measurement**

The crosslinking effect of corilagin is mainly achieved by forming large numbers of H-bonds. They stably mask on the surface of amino groups, and form strongly physical non-covalent interactions, which could avoid potential risk of cytotoxicity caused by other active groups introducing. The number of residual amines on samples were estimate via fixation index (FI) determination and their FI value was exhibited in **Fig. S2**. It was confirmed that corilagin possessed better crosslinking effect. It could construct 3D network within 4h with decellularized tissues, slightly slower than traditional chemical crosslinking agents, but much faster than tissues degradation rate. Then, the final crosslinking degree was higher than 95%, which revealed comparable crosslinking degree to GA and could meet clinical usage needs. In addition, the crosslinking effect of corilagin showed concentration-dependence. As seen, the FI value increased with the concentration of corilagin increasing, reaching its maximum value at a concentration of 1 mg/mL, which was selected as the optimal reactive condition. Therefore, corilagin might be a potential crosslinking reagent for biological tissues fixation in prospective clinical applications. Without introducing other active functional groups, corilagin achieves crosslinking network via large numbers of H-bonds. It could thus avoid continues damage to surrounded cell layer and blood flow.


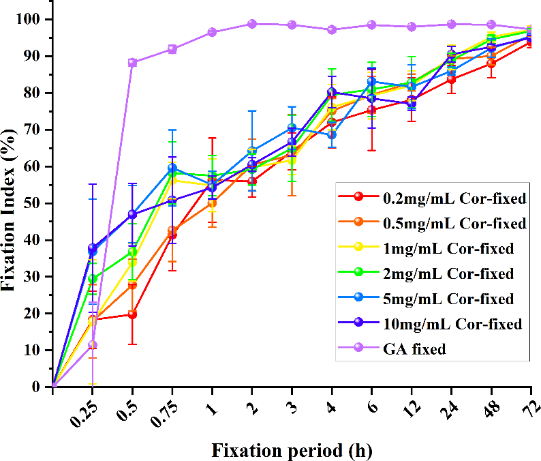


**Fig. S2.** Fixation index (FI) of tissues fixed with a serious concentration of corilagin. All data were calculated from 6 repeated experiments.

**2.5** **Relative weight loss after collagenase and elastase digestion**

The enzymatic hydrolysis period for samples were also extended to 7 days in our research, which are longer than other similar testing. As expected, corilagin crosslinked tissues still exhibited better resistance against enzymolysis (**Fig. S3**). Their weight loss rates are still controlled near 50% after 48 h, while, decellularized-samples are almost completely degraded. Moreover, their enzymatic stability is still concentration-dependent increasing, which indicated we could obtain blood vessels with optimal structural stability and matched mechanical strength. Once the degradation period is extended to 7 days, 2 mg/mL-corilagin crosslinked samples could still maintain over 50% residual quality. It implied that corilagin constructed a dense obstacle to prevent enzymatic penetration into tissues.


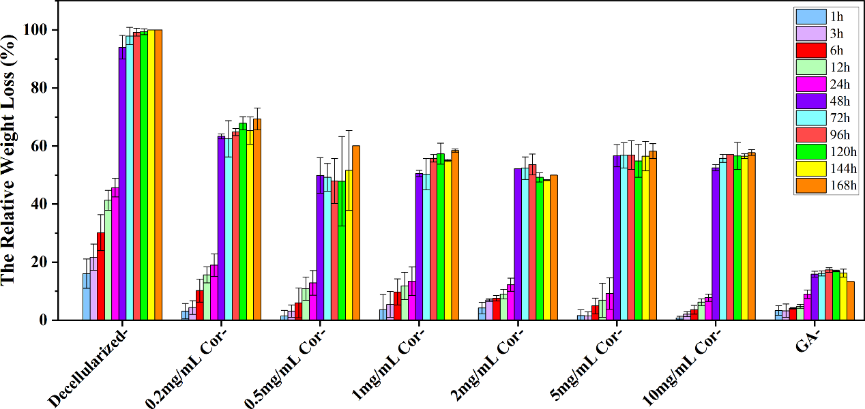


**Fig. S3.** Relative weight of samples after 7 days’ collagenase and elastase digestion. All experimtnes were repeated for at least 3 times.

**2.6 Corilagin releasing**

As well known, corilagin is a highly bioactive small molecule, which has good solubility. If there is no effective crosslinking formed, corilagin will undergo explosive release in a short period and reach a maximum concentration in solution quickly. While, if formed a stable crosslinking, corilagin will be slowly released over a long period. As exhibited below (**Fig. S4**), the release rate of corilagin is very slow, evenly their cumulative releasing amounts are less than 20% after 48 h immersion. It indicated that corilagin has formed stable crosslinking with biological tissues via dense and multiple H-bonds. Moreover, thanks to the biodegradability of tissues, corilagin molecules will be gradually released, with the potential to exert biological activity at vascular anastomosis to maintain blood vessels long-term patency.

.



**Fig. S4.** Corilagin releasing amount (mg/mL) during 48h immersion period. All experiments were repeated at least for 3 times.

**2.7** **Plasma coagulation parameters in vitro**

The effects of corilagin-crosslinking on clotting activity were assayed in vitro via activated partial thromboplastin time (APTT), prothrombin time (PT), thrombin time (TT), and fibrinogen (FIB), which were sensitive for examining blood coagulation state after blood vessels’ implantation. As shown in **Fig. S5**, the clotting activity of all corilagin groups were within normal levels, which indicated that their hydrogen bonding crosslinking does not induce the activation of coagulation mechanisms. Moreover, corilagin crosslinking could significantly prolong APTT (P＜0.05). For TT and PT, compared with the GA-crosslinking (typical chemical method) group, all of corilagin in various concentration could prolong them. It implied samples suffered from corilagin crosslinking could inhibit blood coagulation through exogenous pathway and coagulation pathway. While, compared to them, decellularized group exhibited significantly prolonged PT and TT, as well as a decreased FIB. It could be attributed to that there are trace residual cellular components in the decellularized samples, which may trigger a slight immune reaction and induce hemolysis. Then, the release of components within red blood cells may interfere with their coagulation activity. Subsequent crosslinking modification could further completely remove cellular components and inhibit hemolysis.


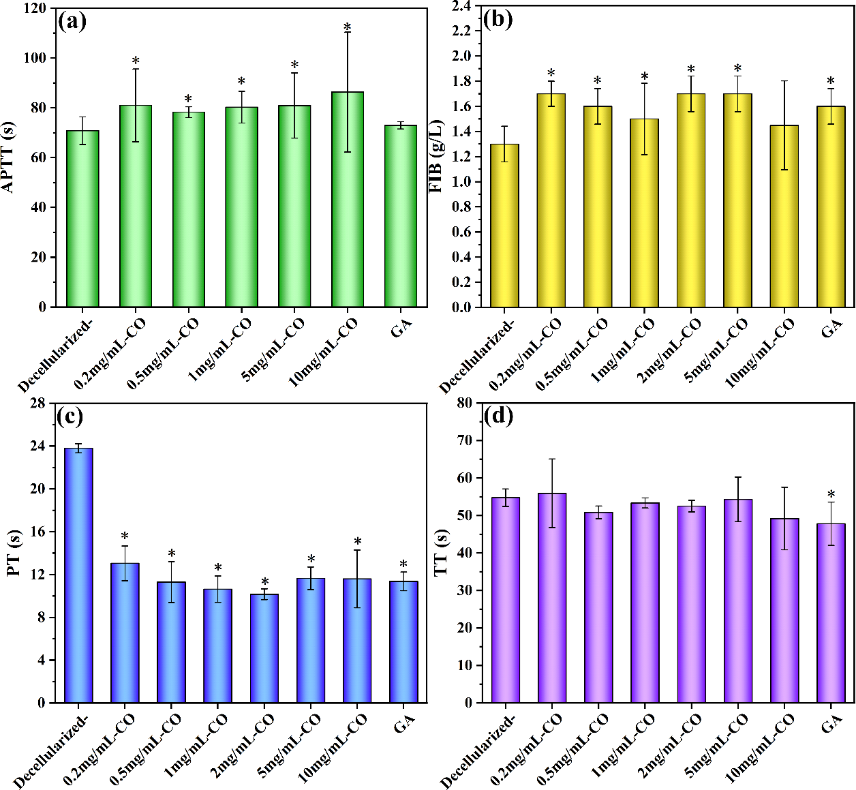


**Fig. S5.** Effects of corilagin-crosslinked samples on plasma coagulation parameters in vitro: (a) APTT, (b) FIB, (c) PT and (d) TT. All experiments were repeated at least for 3 times.

**2.8 Complement activation in vitro**

To detect complement activation by phenol groups, the concentration of C3a and SC5b-9 in whole blood co-incubated with various samples were measured by ELISA assay. In the complement system, C3 is a central component in complement activation, immune defense and immune regulation. In their function process, the critical step is C3 conversion to C3b and C3a, which is responsible to eliminate the pathogen and opsonization. However, complement activation also triggers a series of cascade reactions in vivo, and forms various complement division products, such as C3a and SC5b-9. They could increase vascular permeability, induce various bioactive substances releasing, promote leukocyte aggregation, and even cause serious organ dysfunction. Therefore, we need to determine whether polyphenol groups will cause complement activation. As displayed in **Fig. S6**, the complement concentration is within the normal range after decellularized treatment. Then, corilagin crosslinking will adjust their concentration level slightly, with no significant difference. It indicated that the phenol groups in corilagin are safe for blood vessel crosslinking, which exhibited no complement-activating activity through alternative pathways.


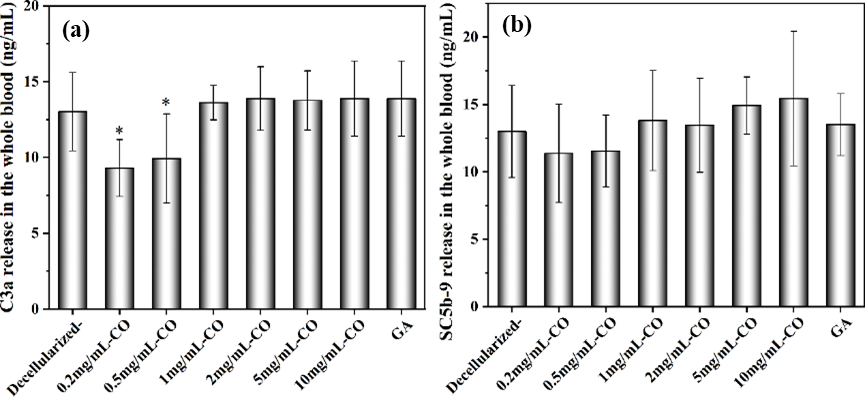


**Fig. S6.** Effects of corilagin-crosslinked samples on complement activation in vitro: (a) C3a and (b) SC5b-9 release in the whole blood. All experiments were repeated at least for 3 times.

**2.9 Hemolysis rate (HR) analysis**

Generally, red blood cell rupture caused by hemolysis may lead to serious anemia, hemoglobinuria, and even acute thrombosis in a short time. Implanted blood vessels will act as a long-term blood-contacting medical device, while, the occurrence of hemolysis can lead to systemic reaction, which may affect the life safety of patients. For dECM-based implants, their rapid degradation and occasional solvent residue tend to induce acute changes in the local biological microenvironment, leading to huge damage to erythrocyte cells. In addition, active functional groups derived from chemical modifications may also react with cell membranes and cause their rupture. Thus, the hemolysis rate (HR) of different samples was quantitatively detected, and purified water and PBS solution were employed as positive (hemolysis) and negative (non-hemolysis) control, respectively. The HR as a function of suspension concentration was evaluated to simulate varying local concentration caused by degradation products. As exhibited in **Fig. 4(e)** and **Fig. S7**, the HR of decellularized- and GA-fixed tissues went up to 20.97% and 13.18% in 4000 μg/mL specimens’ suspension, which were far beyond the safety value (5%) in clinical application. It reflected that they have a higher risk of hemolysis after implantation. It may be attributed to following reasons: free amino groups on decellularized tissues lead to serious immunological rejection, which cause vulnerability and poor corrosion resistance of RBCs. And the high HR value of GA-fixed samples may be related to destruction of the lipid bilayer of RBC caused by unreacted aldehyde groups. Fortunately, acceptable HR values were achieved in corilagin-crosslinked samples at various concentration. It is benefit from the basic protection for dECM substrate provided by crosslinking. It indicated that H-bonds introducing can completely mask the free amino groups on the surface of tissues and avoid their immunological rejection. Meanwhile, it can also avoid local tissues degeneration caused by active groups’ direct chemical reaction with amino groups. Under non-pathological conditions (in a neutral buffer system), the HR of corilagin-fixed groups showed the highest value of 3%, which is far below the international safety standard, indicating a stable and protective vessel environment to avoid the RBC destruction caused by small active molecule.

**
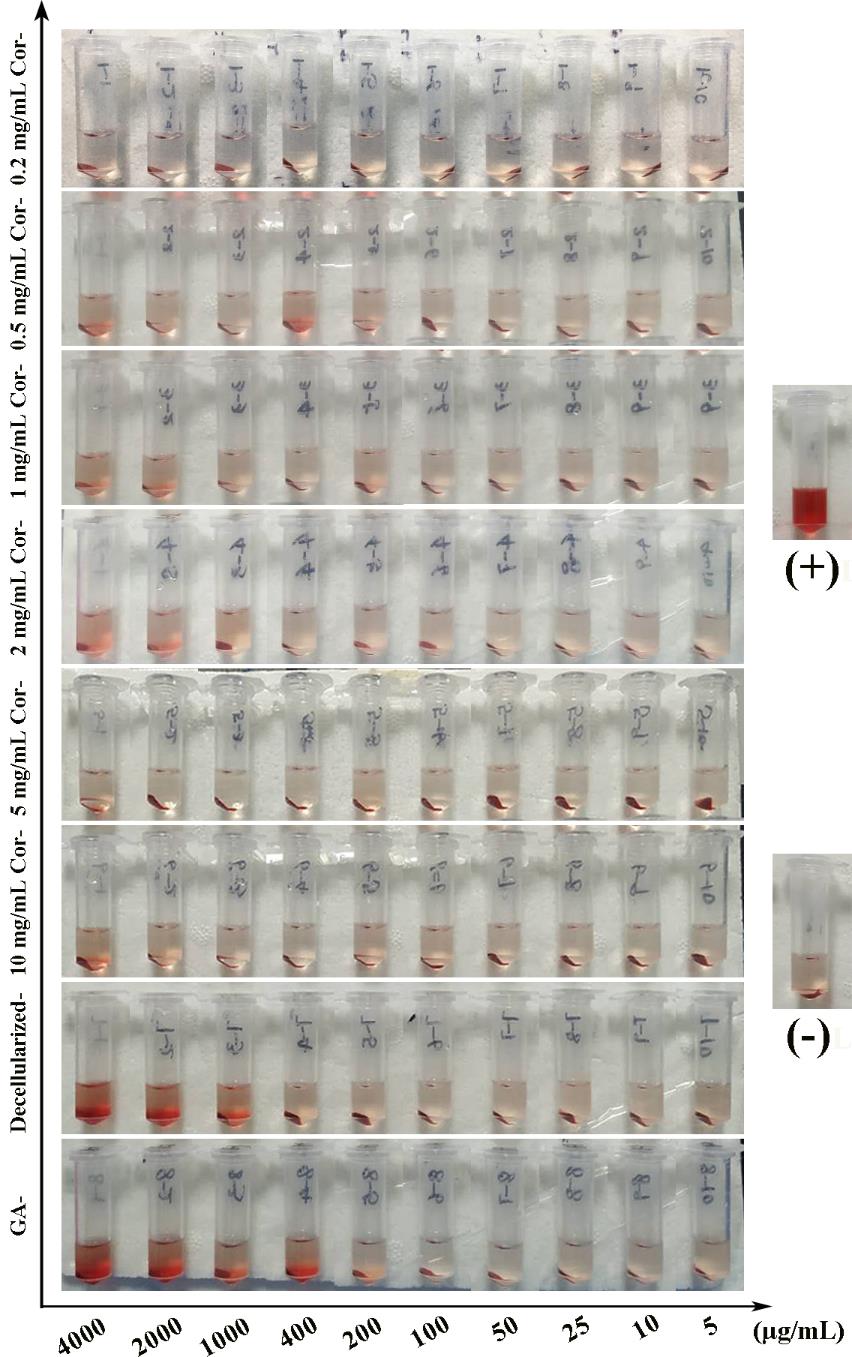
**

**Fig. S7.** Hemolysis photographs as a function of suspension concentration for various samples after incubating with RBCs at 37 ^o^C for 4 h. All these experiments were repeated at least for 3 times.

**2.10 Cell viability of HUVECs**

Endothelial cells will form a complete protective layer on the surface of blood vessels, which is considered as important prerequisites for the function of them. Therefore, cell viability of HUVECs on various samples fixed by corilagin were detected by CCK-8 measurement. The results were exhibited in **Fig. S8**.

**
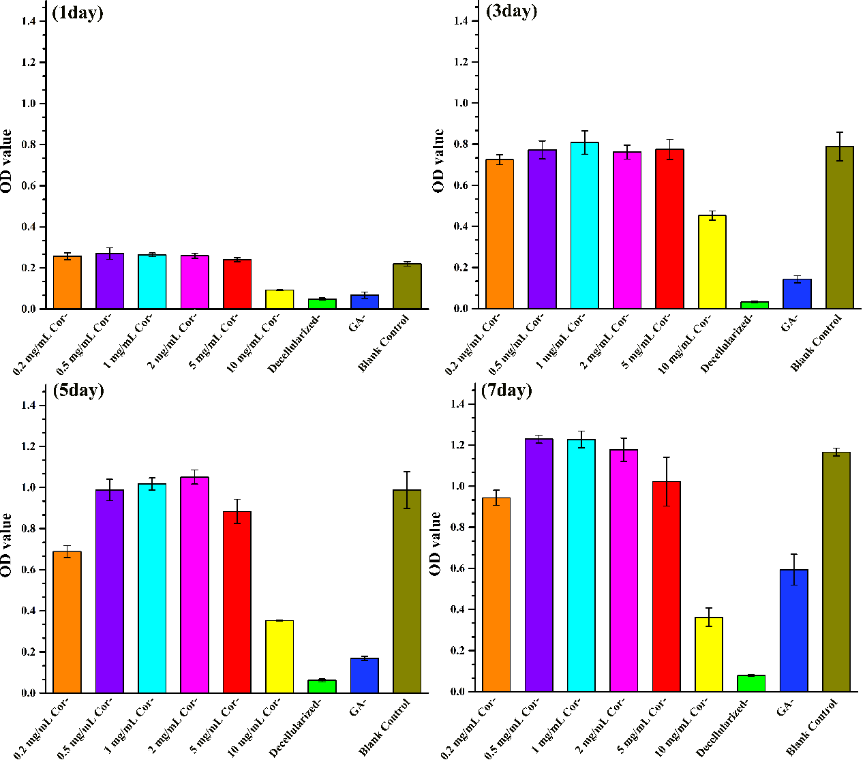
**

**Fig. S8.** Cell viability of HUVECs on various samples measured by CCK-8 after 1 days, 3 days, 5 days, and 7 days co-culture. All these experiments were repeated at least for 3 times.

HUVECs possessed excellent proliferation on corilagin-treated tissues. In addition, corilagin’s promoting effect showed significant concentration dependence: With the concentration of corilagin increased, HUVECs viability gradually increased, and reached its peak value at about 1 mg/mL concentration. It revealed that corilagin-treated samples provided a better growth environment for HUVECs, and 1 mg/mL was selected as the optimal concentration for promoting cell viability. While, on the seventh day, cells proliferation is inhibited on the contrary, due to excessive cell count and cells spreading in limited space. Similar result was obtained in SEM observation: No visible normally adherent cell could be identified on GA-fixed samples, few scattered ones exhibited small spherical shape, representing non-adherent morphology and high cytotoxicity. It further indicated that corilagin crosslinking not only can absolutely eliminate the immunogenicity of natural biological tissues, but also avoid potential cytotoxicity caused by other active functional groups introducing.

**2.11 ROS analysis for inflammatory risk**

The production of excessive ROS at anastomotic site is the main reason for chronic inflammation, and even acute thrombosis. For blood vessels implantation, persistent infection can lead to excessive ROS accumulation, and the activated immune system can also produce a large number of ROS, all of which cause a significant increase in oxidative stress in microenvironment. Owing to large numbers of phenol groups in corilagin, the crosslinked blood vessels are expected to exhibit promising anti-oxidation effect for vascular anastomosis healing. In this case, their in vivo anti-oxidative efficiency was investigated by flow cytometry analysis (FAC). As exhibited in **Fig. S9**, ROS level showed obvious concentration-dependence: The ROS level decreased gradually with corilagin concentration increasing, and reached a minimum at 2 mg/mL, which is benefitted to maintain ROS homeostasis and help alleviate oxidative stress. Especially, the ROS analysis in FAC indicated that 2 mg/mL corilagin-crosslinked samples presented obvious oxidation resistance with 80% radical scavenging efficiency. Notably, samples all exhibited similar antioxidant activity once the concentration was over 5 mg/mL, or even slightly decreased, without more outstanding radical scavenging property. During this measurement, dichlorodihydro-fluorescein diacetate (DCFH-DA) was used as a probe to detect the ROS level.


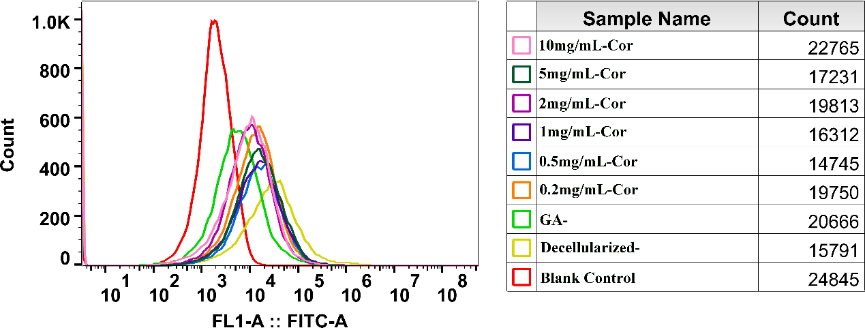


**Fig. S9.** ROS analysis for inflammatory risk

**2.12 IHC analysis for inflammatory response in vivo**

For natural derived blood vessels, immune response is one of the most critical problems to cause failure implantation. Especially, some inflammation-related factors secretion may lead to acute thrombosis, stenosis and calcification. Therefore, TNF-α, as a typical pro-inflammatory factor, was detected via ELISA measurement.

As **Fig. S10** shown, decellularized-samples exhibited a significant pro-inflammatory tendency, which may be attributed to persistent toxicity caused by dissolved degraded substances in tissues cavities. In addition, although GA formed a dense crosslinking network in tissues to prevent degradation, strong positive staining for inflammatory indicators (TNF-α) were observed. That may because the overflow of dissolved toxic GA monomer changes the capillary permeability of normal blood vessels, which caused some inflammatory-related factors releasing and increased their local concentration. On the contrary, TNF-α secretion from corilagin-crosslinked tissues were suppressed, which were also proved by other pro-inflammatory or anti-inflammatory factors release regulation in **Fig. 6**. Besides, this process exhibited concentration-dependence. That is, with the concentration of corilagin increasing, the inhibitory effect on TNF-α secretion is enhanced and reaches a peak value at 1 mg/mL, which was selected as the optimal concentration in subsequent test. However, how do they achieve this anti-inflammatory function is still unknown.

**
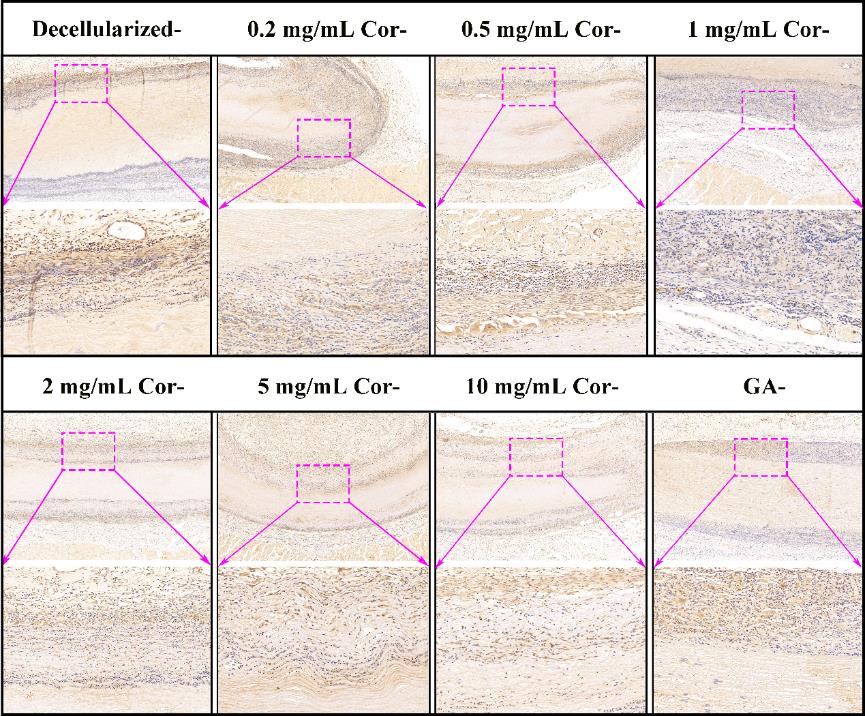
**

**Fig. S10.** IHC analysis for inflammatory response in vivo with antibody to TNF-α after 4-weeks subcutaneous implantation

**2.13 In vivo mineral deposition**

Generally, osteocalcin (OCN) is widely spread in various bone structures (such as bone and teeth) in vivo. It is regarded as a sign of osteoblast differentiation and maturation and plays an important role in the process of mineralization in vivo. In normal soft tissues, such as healthy blood vessels, OCN expression was almost undetectable. However, once calcification deposition occurs, it will have a high expression in local. At the same time, fibers stiffness and local tissues degeneration will be observed. Therefore, OCN secretion was regarded as an important early indicator of vascular calcification in pathological changes. As **Fig. S11** exhibited, there are serious calcification on decellularized- and GA-fixed samples. That may because their residual immunogenicity and degradation products act as calcification sites in vivo. While, once corilagin introduced, only little calcification or tiny number of immune responses were detected. It suggested that polyphenol compounds such as corilagin may effectively inhibit the secretion of inflammatory-related factors, thus avoid calcification genes activation. Besides, the inhibitory effect of corilagin on vascular calcification showed an obvious concentration-dependence regulation. As the concentration of corilagin increased, the number of mineral depositions decreased. It reached an optimal effect on 1 mg/mL, and there are almost no calcification points on its surface. Similar result was also obtained in **Fig. 11**.


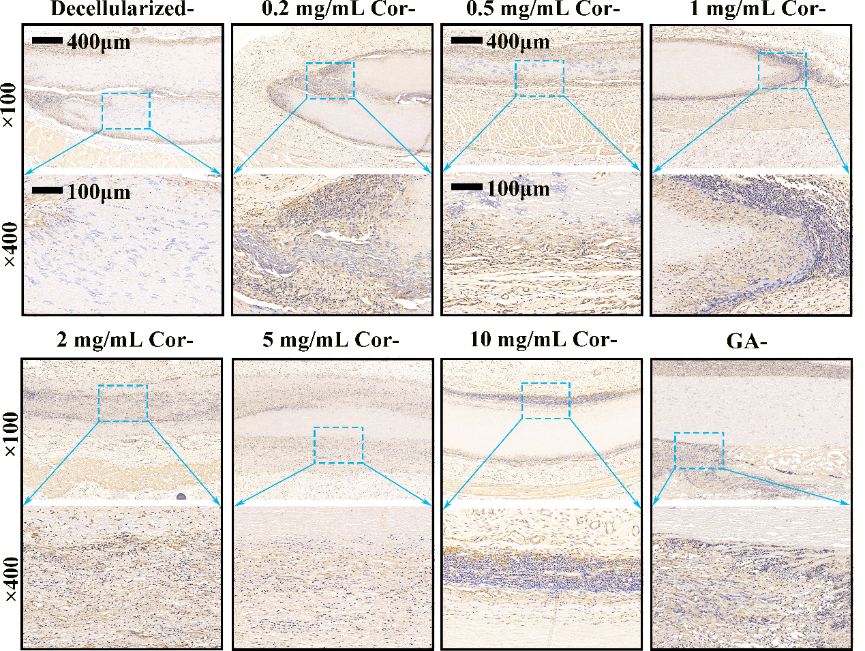


**Fig. S11.** Images of in vivo mineral deposition stained with OCN after 12-weeks subcutaneous implantation in SD rats.
